# Derivatives of 2-Pyridone Exhibiting Hot-Exciton TADF for Sky-Blue and White OLEDs

**DOI:** 10.1021/acsaelm.3c00443

**Published:** 2023-07-21

**Authors:** Iryna Danyliv, Khrystyna Ivaniuk, Yan Danyliv, Igor Helzhynskyy, Viktorija Andruleviciene, Dmytro Volyniuk, Pavlo Stakhira, Glib V. Baryshnikov, Juozas V. Grazulevicius

**Affiliations:** † Lviv Polytechnic National University, Stepan Bandera 12, 79013 Lviv, Ukraine; ‡ Department of Polymer Chemistry and Technology, Kaunas University of Technology, K. Barsauskas str. 59, Kaunas 51423, Lithuania; § Laboratory of Organic Electronics, Department of Science and Technology, Linköping University, SE-60174 Norrköping, Sweden; ∥ Department of Chemistry and Nanomaterials Science, Bohdan Khmelnytsky National University, 18031 Cherkasy, Ukraine

**Keywords:** pyridone, hot exciton, delayed fluorescence, exciplex, OLED

## Abstract

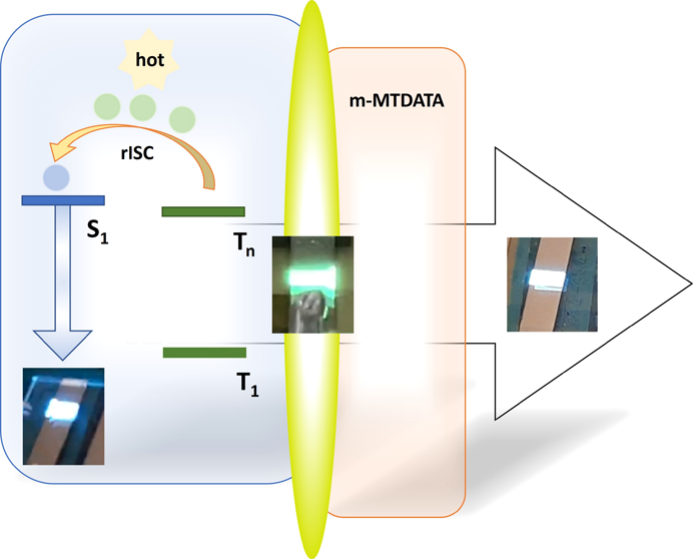

Development of emissive materials for utilization in organic light-emitting diodes (OLEDs) remains a highly relevant research field. One of the most important aspects in the development of efficient emitters for OLEDs is the efficiency of triplet-to-singlet exciton conversion. There are many concepts proposed for the transformation of triplet excitons to singlet excitons, among which thermally activated delayed fluorescence (TADF) is the most efficient and widespread. One of the variations of the TADF concept is the hot exciton approach according to which the process of exciton relaxation into the lowest energy electronic state (internal conversion as usual) is slower than intersystem crossing between high-lying singlets and triplets. In this paper, we present the donor–acceptor materials based on 2-pyridone acceptor coupled to the different donor moieties through the phenyl linker demonstrating good performance as components of sky-blue, green-yellow, and white OLEDs. Despite relatively low photoluminescence quantum yields, the compound containing 9,9-dimethyl-9,10-dihydroacridine donor demonstrated very good efficiency in sky-blue OLED with the single emissive layer, which showed an external quantum efficiency (EQE) of 3.7%. It also forms a green-yellow-emitting exciplex with 4,4′,4″-tris[phenyl(*m*-tolyl)amino]triphenylamine. The corresponding OLED showed an EQE of 6.9%. The white OLED combining both exciplex and single emitter layers demonstrated an EQE of 9.8% together with excellent current and power efficiencies of 16.1 cd A^–1^ and 6.9 lm W^–1^, respectively. Quantum-chemical calculations together with the analysis of photoluminescence decay curves confirm the ability of all of the studied compounds to exhibit TADF through the hot exciton pathway, but the limiting factor reducing the efficiency of OLEDs is the low photoluminescence quantum yields caused mainly by nonradiative intersystem crossing dominating over the radiative fluorescence pathway.

## 1 Introduction

Rapid development of science and technology requires development of new organic materials and devices based on them as components of solid-state lighting and display technologies, medicine, biotechnology, etc. Considerable research interest in the development of new organic semiconductors for organic light-emitting devices (OLEDs) is related to the applications of these devices as components of flat panel displays, mobile electronics, smartphones, and lighting devices.^1−3^ Despite the continuous efforts in the development of improved materials and devices, there is still room for their perfection.

A convenient way to improve device efficiency is the utilization of emitters exhibiting delayed fluorescence (DF). DF can be realized by the utilization of triplet excitons through the upconversion of nonradiative triplet states into radiative singlet states^4^ in two ways. Two excited triplet states (*T*_1_) during the upconversion process can produce one singlet (*S*_1_) excited state. This process is named triplet–triplet annihilation (TTA). In another way, the temperature-assisted reverse intersystem crossing (RISC) process results in 100% efficiency of triplet state harvesting.^5^ The process is known as thermally activated delayed fluorescence (TADF). The TADF mechanism appears by the conversion of harvested triplet excitons from the locally excited triplet state *T*_1(LE)_ to the locally excited singlet state *S*_1(LE)_ at the same spectral wavelength.^6^ Small singlet–triplet energy splitting (Δ*E*_S-T_), spatial separation of highest occupied molecular orbital (HOMO)/lowest unoccupied molecular orbital (LUMO) with a small overlap, and efficient RISC process are the main requirements for the realization of the TADF mechanism.^7,8^ RISC can occur not only between *T*_1_ and *S*_1_ but also between higher triplet (*T_n_*, *n* ≥ 2) and singlet states (*S_m_*, *m* ≥ 1).^9^ Compounds allowing triplet harvesting via upper-level triplet–singlet RISC are used as emitters and hosts in the fabrication of efficient host-free OLEDs with an external quantum efficiency (EQE) higher than 5%, which is the theoretical limit of EQE for electroluminescent devices based on emitters exhibiting prompt fluorescence.^10−13^ Consequently, one of the ways of further enhancement of efficiency of the OLEDs is related to the development of novel compounds, including those exhibiting TADF, which occurs via upper-level triplet–singlet RISC.

Electron-donating nitrogen-containing aromatic heterocyclic compounds such as 9*H*-carbazole, 9,9-dimethyl-9,10-dihydroacridine, 9*H*-phenothiazine, 9*H*-phenoxazine, etc. have been widely used in the synthesis of efficient TADF emitters.^14−16^ Their derivatives are characterized by high thermal and electrochemical stability, good hole-transporting properties, and high and stable triplet states.^17−19^ In turn, electron-withdrawing moieties, such as nitrile,^20,21^ triazine,^22,23^ pyrimidine,^24,25^ pyridine,^26^ etc. were widely applied in the design of bipolar TADF emitters. For the achievement of TADF, careful selection of the combination of donor (D) and acceptor (A) moieties in the molecules is required.

In this article, we report the synthesis and characterization of new D–A-type TADF emitters based on a small acceptor (pyridin-2(1*H*)-one) moiety combined with a series of common donor species (9*H*-carbazole, 9,9-dimethyl-10*H*-acridane, 9*H*-phenothiazine, and 9*H*-phenoxazine). The compound containing 9,9-dimethyl-10*H*-acridane moiety, which exhibited the highest photoluminescence quantum yield in the solid state, was tested in electroluminescent devices. The EQE values of 3.7, 6.9, and 9.8% were achieved for sky-blue OLED with the single emissive layer, for green-yellow OLED based on the emission of exciplex with 4,4′,4″-tris[phenyl(*m*-tolyl)amino]triphenylamine (MTDATA), and for white OLED based on the combination of both exciplex and single layer emissions, respectively. In addition, this compound demonstrated the effect of “hot” exciton TADF.^9^

## 2 Results and Discussion

### 2.1 Molecular Design and Synthesis

**Scheme 1:**
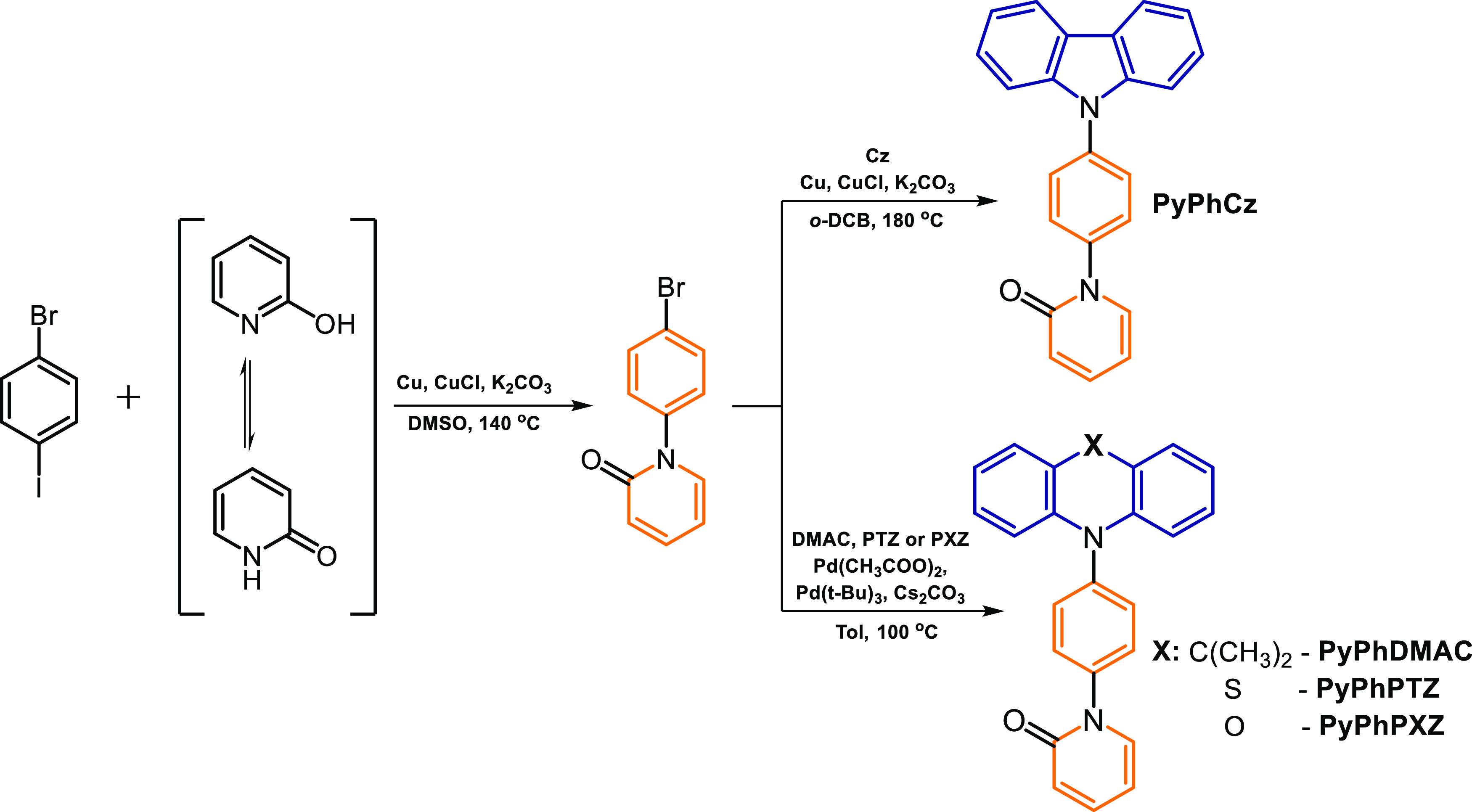
Synthetic Routes and Chemical Structures of Target Compounds

It is well known that 2-hydroxypyridine is capable of exhibiting keto–enol tautomerization under specific conditions.^27^ Utilization of this property makes it possible to implement two consequent amination reactions, which are depicted in Scheme 1. 9*H*-Carbazole, 9,9-dimethyl-9,10-dihydroacridine, 9*H*-phenothiazine, and 9*H*-phenoxazine were selected for the molecular design of the target compounds. The first step in the synthesis was Ullmann condensation, which afforded 2-pyridone substituted with a bromophenyl moiety. The following Ullmann condensation (in case of 9*H*-carbazole aromatic amine) or Buchwald–Hartwig cross-coupling reaction (in case of 9,9-dimethyl-9,10-dihydroacridine, 9*H*-phenothiazine and 9*H*-phenoxazine aromatic amines) yielded the target compounds 1-(4-(9*H*-carbazol-9-yl)phenyl)pyridin-2(1*H*)-one (**PyPhCz**), 1-(4-(9,9-dimethylacridin-10(9*H*)-yl)phenyl)pyridin-2(1*H*)-one (**PyPhDMAC**), 1-(4-(10*H*-phenothiazin-10-yl)phenyl)pyridin-2(1*H*)-one (**PyPhPTZ**), and 1-(4-(10*H*-phenoxazin-10-yl)phenyl)pyridin-2(1*H*)-one (**PyPhPXZ**). The chemical structures of the obtained derivatives were confirmed by ^1^H and ^13^C NMR spectroscopy, mass spectrometry, and elemental analysis (Supporting Information).

### 2.2 Thermal Characterization

**Figure 1:**
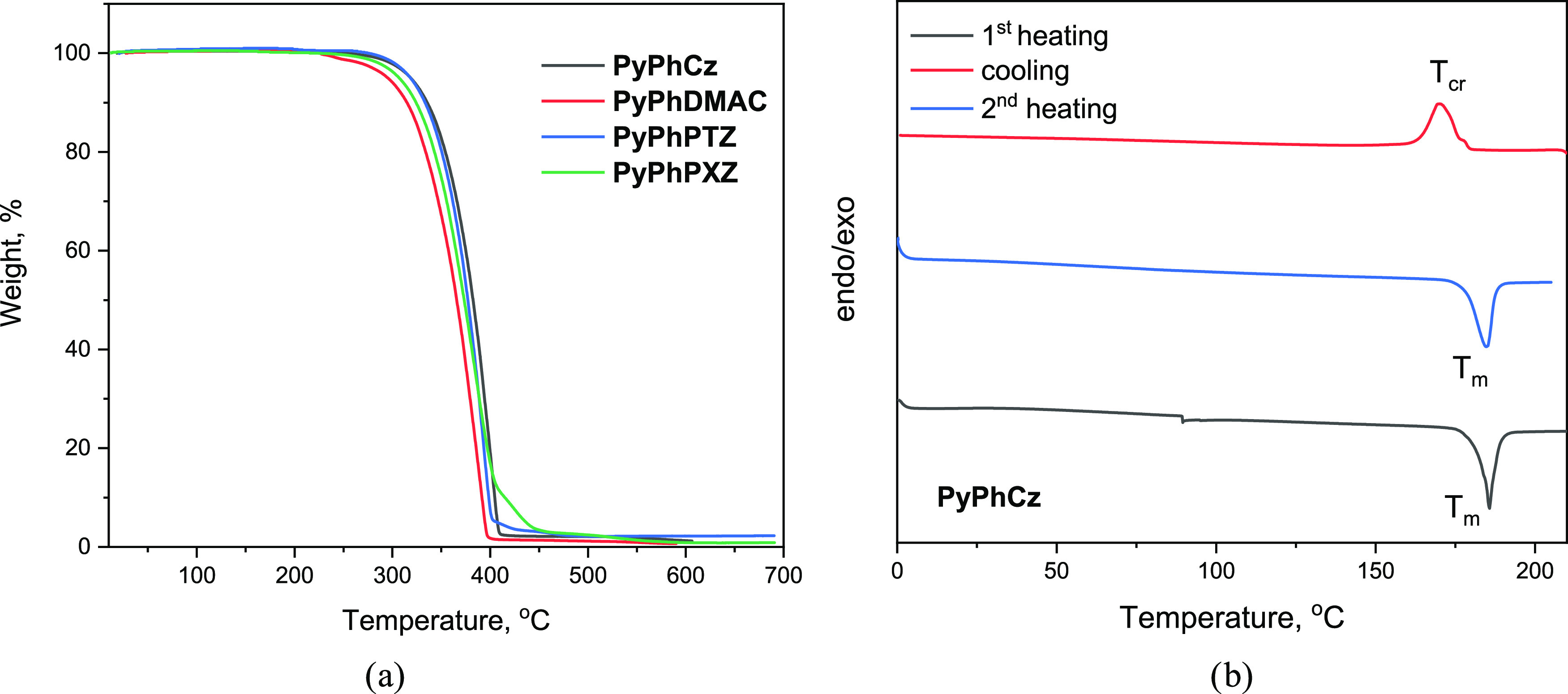
(a) TGA of the derivatives of 2-pyridone and (b) DSC curves of **PyPhCz**.

The thermal properties of the synthesized compounds were investigated by thermogravimetric analysis (TGA) and differential scanning calorimetry (DSC). TGA and DSC curves are shown in Figures 1a,b and S1. The temperatures of the transitions are summarized in Table 1. All derivatives exhibited a relatively high temperature of 5% weight loss (*T*_ID_) ranging from 295 to 320 °C. The single-stage TGA curves reflecting full weight loss show that most of the compounds are subjected to sublimation during TGA measurements. The 2-pyridone derivatives were obtained as crystalline substances after synthesis and purification. Therefore, the endothermic melting peaks were observed for all of the derivatives in the first DSC heating scans (Figures 1b and S1). In the following cooling scans, compounds **PyPhCz** and **PyPhDMAC** showed crystallization signals at 170 and 167 °C, respectively. The melting signals were observed in the subsequent heating scans at 185 and 247 °C (Figures 1b and S1, Table 1). Meanwhile, compounds **PyPhPTZ** and **PyPhPXZ** were found to be able to form molecular glasses with glass-transition temperatures (*T*_g_) of >66 °C (Figure S1 and Table 1).

**Table 1:** Thermal Characteristics of the Derivatives of 2-Pyridone

compound	*T*_ID_^a^, °C	*T*_m_^b^, °C	*T*_cr_^c^, °C	*T*_g_^d^, °C
**PyPhCz**	320	185	170	–
**PyPhDMAC**	295	247	167	–
**PyPhPTZ**	318	206	–	66
**PyPhPXZ**	307	175	–	66

a Determined by TGA; determined by DSC from.

b First heating scan.

c Cooling.

d Second heating scan.

### 2.3 Photophysical Properties

**Figure 2:**
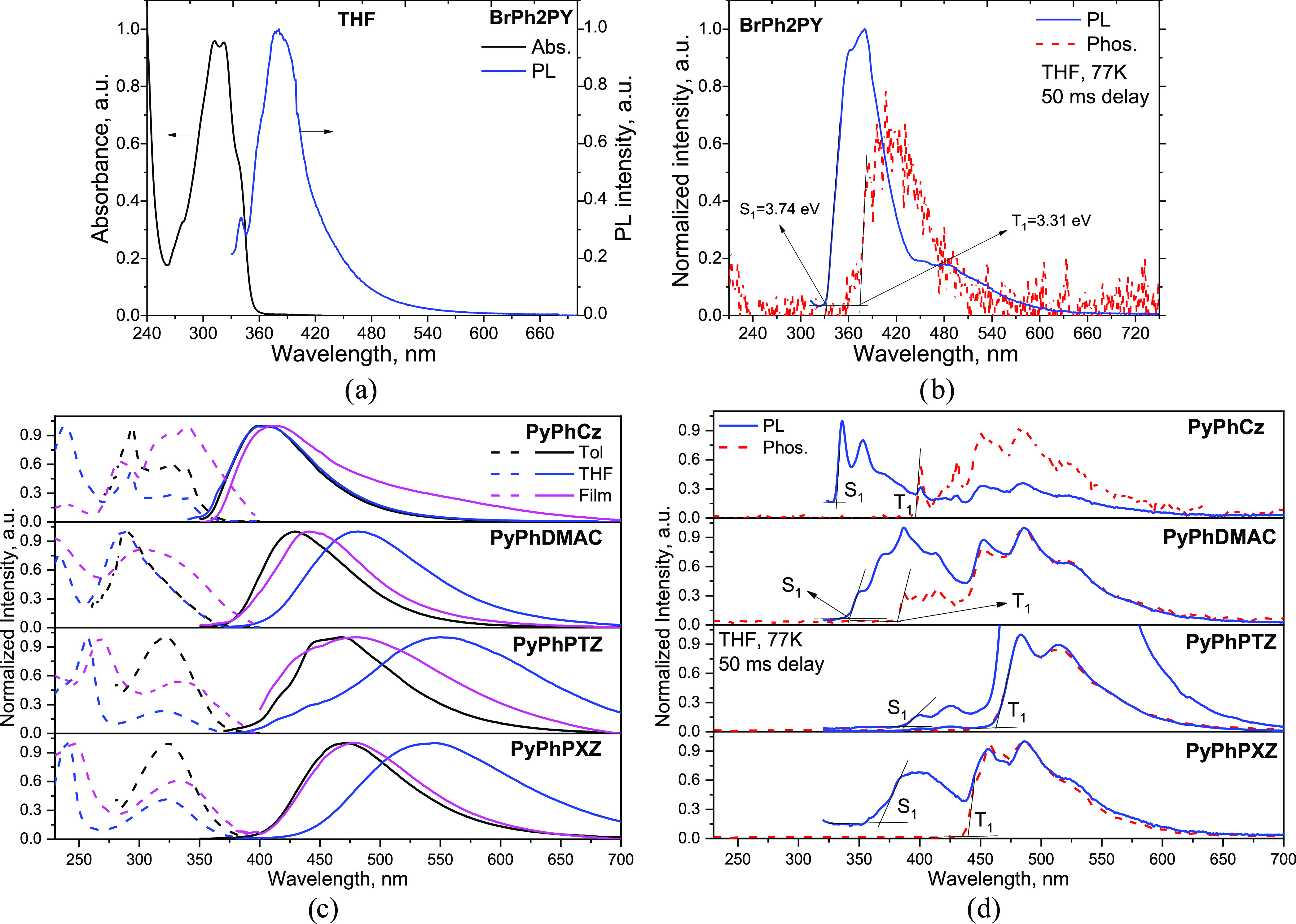
Absorption and PL spectra (a) as well as PL and Phos. spectra (b) of the dilute THF solution of BrPh2PY recorded at 77 K. Absorption (dashed lines) and PL (solid lines) spectra (c) of the toluene (Tol) and THF solutions as well as of solid films (Film) of the derivatives of 2-pyridone and PL and Phos. spectra (d) of their dilute THF solutions recorded at 77 K.

Before the investigation of the photophysical properties of the target compounds, UV–vis absorption and photoluminescence (PL) spectra of the tetrahydrofuran (THF) solution of acceptor 1-(4-bromophenyl)pyridin-2(1*H*)-one (BrPh2PY) were recorded (Figure 2a). In addition, PL and phosphorescence (phosphorus) spectra of the THF solution of BrPh2PY were recorded at 77 K (Figure 2b). The low-energy absorption band of the dilute THF solution of BrPh2PY peaked at 312 and 322 nm. The PL spectrum of BrPh2PY has a maximum at 379 nm. The relatively high lowest singlet (*S*_1_ = 3.74 eV) and triplet (*T*_1_ = 3.31 eV) energy levels were obtained for the selected acceptor (BrPh2PY). Typically, electron-accepting and electron-donating moieties with high triplet levels are utilized in the design of efficient TADF emitters.^28^ Analysis of the photophysical properties of BrPh2PY shows that it has great potential to be used in the design of TADF emitters with different emission colors, including deep-blue color.

UV–vis absorption and photoluminescence (PL) spectra of the solutions of the derivatives of 2-pyridone in the solvents of the different polarities and of the solid films are shown in Figure 2c,d. The wavelengths of low-energy absorption and PL maxima are collected in Table 2. The low-energy absorption bands of the dilute solutions were observed in the region of 287–340 nm. Taking into account the absorption spectrum of BrPh2PY, it can be presumed that the low-energy absorption bands of the studied derivatives of 2-pyridone (**PyPhs**) result from their donor–acceptor structure. At wavelengths longer than 350 nm, absorption spectra of **PyPhs** are characterized by shoulders with long tails reaching 400 nm. These tails are caused by the intramolecular charge-transfer (CT) states formed by the donor–acceptor interactions in the ground state. The low-energy absorption bands of the solid films were found to be slightly bathochromically shifted (304–380 nm) in comparison with those of the corresponding solutions of the derivatives. The shifts are probably caused by the intermolecular interactions in the solid state.

**Table 2:** Photophysical Characteristics of the Compounds

compound	λ_abs_^a^,^b^,^c^, nm	λ_PL_^a^,^b^,^c^, nm	Φ_PL_^a^	Φ_PL_^b^	Φ_PL_^c^	*S*_1_, eV	*T*_1_, eV	Δ*E*_ST_, eV
**PyPhCz**	340/340/380	399/407/411	0.04	0.02	0.02	3.77	3.12	0.65
**PyPhDMAC**	290/287/304	429/482/441	0.05	0.045	0.04	3.63	3.24	0.39
**28PyPhPTZ**	322/319/336	468/550/480	0.01	0.01	<0.01	3.19	2.67	0.52
**PyPhPXZ**	324/322/333	472/545/480	0.07	0.03	<0.01	3.38	2.82	0.56

a Toluene solution.

b THF solution.

c Solid state. λ_abs_ is the wavelength of absorption maximum; λ_PL_ is the wavelength of photoluminescence maximum; Φ is photoluminescence quantum yield. The concentration of solutions was 10^–5^ M.

The solutions in toluene and thin films of 2-pyridone derivatives demonstrated PL in the violet-blue range of the visible spectrum with emission intensity maxima in the regions of 399–472 and 411–480 nm, respectively. The bathochromic shifts of emission bands were observed for the THF solutions relative to those of the toluene solutions of the derivatives, with the wavelength maximum emission intensities varying from 407 to 550 nm (Table 2). PL and phosphorescence spectra were recorded at 77 K (Figure 2d). The lowest singlet (*S*_1_) and triplet (*T*_1_) energy levels and Δ*E*_ST_ values were determined from the high-energy onsets of the corresponding spectra (Table 2). Large Δ*E*_ST_ values for **PyPhs** (>0.39 eV) do not support the possibility of the reverse intersystem crossing between the lowest triplet and singlet states. The photoluminescence efficiencies were found to be rather low for both, toluene solutions and solid films. The highest photoluminescence quantum yields (PLQY) were observed for the solid sample of **PyPhDMAC** and for a toluene solution of **PyPhPXZ**. Taking into account these observations, we decided to focus on compound **PyPhDMAC** as an emissive component of OLEDs. Surprisingly, **PyPhDMAC**-based **OLED** demonstrated a quite high EQE of 3.7% (more details are given in the next section), which is close to the theoretical limit of 5% for fluorescent OLEDs. We assume that compound **PyPhDMAC** might exhibit TADF. As follows from our quantum-chemical calculations, **PyPhDMAC** sustains a three-state model (Tables 3 and 4) typical for emitters demonstrating TADF of type I through the spin–vibronic coupling mechanism (following nomenclature by Monkman and coauthors).^29^ One should note that the *S*_1_ state of **PyPhDMAC** can be assigned to the charge-transfer (^1^CT) state (HOMO is localized on the donor part, while LUMO is on the acceptor part, Figure S2), while the *T*_1_ state is localized on the acceptor part only (i.e., is the ^3^LE state). Thus, spin–orbit coupling matrix element (SOCME) between these states is considerable (0.64 cm^–1^, Table 4) in line with earlier reported conclusions by Brédas et al.^30^ and is also consistent with the classical El-Sayed rule for ISC rates. The electronic configuration of the *T*_2_ state is the same as for the *S*_1_ state (i.e., *T*_2_ is the ^3^CT state), and thus it is quasi-degenerated with ^1^CT. Therefore, the mechanism of TADF exhibited by **PyPhDMAC** can be considered since ^3^LE is coupled with ^3^CT by vibronic coupling and ^3^CT-to-^1^CT rISC is thus possible (SOCME is small, 0.04 cm^–1^, but nonzero). However, based on the *T*_1_ state geometry of **PyPhDMAC**, our calculations predict very large *S*_1_-*T*_1_ (1.12 eV) and *T*_2_-*T*_1_ (1.11 eV) gaps that unbales efficient reverse internal conversion between ^3^LE and ^3^CT states. Thus, vibronically assisted TADF is not the case for **PyPhDMAC**. Another idea of how TADF can be realized in **PyPhDMAC** is the hot exciton concept.^9^ Accounting for the fact that *S*_1_ and *T*_2_ states are quasi-degenerated and both correspond to HOMO–LUMO electronic configuration, the corresponding ^3^CT (*T*_2_) and ^1^CT (*S*_1_) excitons can be populated initially through electron–hole recombination (OLED is fabricated following the closest energy matching between injected electrons and holes vs LUMO and HOMO energy levels, respectively). Taking into account the very large *T*_2_–*T*_1_ separation, the internal conversion from *T*_2_ to *T*_1_ should be slower than *T*_2_–*S*_1_ reverse ISC (*S*_1_–*T*_2_ gap is only 0.01 eV, SOCME is small but nonzero, 0.04 cm^–1^), which converts *T*_2_ excitons to emissive *S*_1_ excitons. Thus, the TADF channel for *T*_2_ “hot” excitons should dominate over nonradiative quenching of *T*_2_ excitons, which results in the OLED heterostructure demonstrating an external quantum efficiency of around 3.7%. For compound **PyPhCz**, four triplet states are lower in energy than the *S*_1_ state, which makes a triplet yield high and thus quenches the *S*_1_ state fluorescence. The rISC channel for compound **PyPhCz** is also possibly accounting for the close-lying *T*_4_ and *S*_1_ states of the same HOMO–LUMO configuration coupled by SOC with ⟨*S*_1_|**Ĥ**_**SO**_|*T*_*n*_⟩ = 0.20 cm^–1^ (at *T*_1_ geometry). Also the *T*_5_–*S*_2_ channel is a possible way to get rISC in **PyPhCz**. The gap is less than 0.1 eV, and the SOCME is 0.23 cm^–1^.

**Table 3:** Excited State Energies for the Derivatives of 2-Pyridone^a^

compound	*E*(*S*_1_)vert	*E*(*S*_1_)^S1^	*E*(*T_n_*)^S1^	*E*(*T*_1_)vert	*E*(*S*_1_)^T1^	*E*(*T_n_*)^T1^
**PyPhCz**	3.77 (0.36) [3.65]	3.28 (0.38) [3.11]	2.30 (*n* = 1)	2.73	3.67	2.59 (*n* = 1)
			3.00 (*n* = 2)			2.73 (*n* = 2)
			3.24 (*n* = 3)			3.07 (*n* = 3)
						3.43 (*n* = 4)
**PyPhDMAC**	3.36 (10^–4^)	2.92 (10^–3^) [2.89]	2.30 (*n* = 1)	2.73	3.11	1.99 (*n* = 1)
			2.91 (*n* = 2)			3.10 (*n* = 2)
**PyPhPTZ**	3.41 (5 × 10^–4^)	2.654 (4 × 10^–4^) [2.65]	2.29 (*n* = 1)	2.73	2.58	1.94 (*n* = 1)
			2.41 (*n* = 2)			2.73 (*n* = 2)
			2.648 (*n* = 3)			2.98 (*n* = 3)
**PyPhPXZ**	3.09 (2 × 10^–4^)	2.579 (4 × 10^–4^) [2.63]	2.30 (*n* = 1)	2.73	2.84	1.99 (*n* = 1)
			2.54 (*n* = 2)			2.77 (*n* = 2)
			2.577 (*n* = 3)			2.82 (*n* = 3)

a Calculated by the TDDFT/LC-ωPBE/6-31G(d) method (ω = 0.14; PCM model was used for accounting of toluene solvent effect). Experimental references are presented in parentheses.

**Table 4:** Orbital Nature of Low-Lying Excited States for the Derivatives of 2-Pyridone Together with the Values of Spin–Orbit Coupling Matrix Elements (SOCMEs) ⟨*S*_1_|**Ĥ**_**SO**_|*T*_*n*_⟩ Calculated at *S*_1_ and *T*_1_ State Geometries

compound	⟨*S*_1_|**Ĥ**_**SO**_|*T*_*n*_⟩^S1^, cm^–1^	⟨*S*_1_|**Ĥ**_**SO**_|*T*_*n*_⟩^T1^, cm^–1^	assignment^S1^	assignment^T1^
**PyPhCz**	0.30 (*n* = 1)	0.04 (*n* = 1)	S_1_: H → L (96%)	S_1_: H → L (89%)
	0.75 (*n* = 2)	0.53 (*n* = 2)	T_1_: H-2 → L (78%)	T_1_: H → L+1 (73%)
	0.30 (*n* = 3)	0.08 (*n* = 3)	T_2_: H → L (61%)	T_2_: H-2 → L (72%)
		0.20 (*n* = 4)	T_3_: H-1 → L (88%)	T_3_: H-1 → L+1 (72%)
				T_4_: H → L (65%)
**PyPhDMAC**	0.93 (*n* = 1)	0.64 (*n* = 1)	S_1_: H → L (97%)	S_1_: H → L (97%)
	0.10 (*n* = 2)	0.04 (*n* = 2)	T_1_: H-1 → L (86%)	T_1_: H-1 → L (90%)
			T_2_: H → L (97%)	T_2_: H → L (96%)
**PyPhPTZ**	0.69 (*n* = 1)	3.21 (*n* = 1)	S_1_: H → L (98%)	S_1_: H → L+1 (93%)
	1.22 (*n* = 2)	1.75 (*n* = 2)	T_1_: H-1 → L (75%)	S_2_: H → L (70%)
	0.10 (*n* = 3)	2.96 (*n* = 3)	T_2_: H → L+1 (69%)	T_1_: H → L+1 (88%)
			T_3_: H → L (95%)	T_2_: H-1 → L (62%)
				T_3_: H → L (68%)
**PyPhPXZ**	0.75 (*n* = 1)	0.58 (*n* = 1)	S_1_: H → L (98%)	S_1_: H → L (97%)
	1.35 (*n* = 2)	1.39 (*n* = 2)	T_1_: H-1 → L (85%)	T_1_: H-1 → L (90%)
	0.13 (*n* = 3)	0.09 (*n* = 3)	T_2_: H → L+3 (49%)	T_2_: H → L+3 (58%)
			T_3_: H → L (94%)	T_3_: H → L (96%)

Similarly, compound **PyPhPTZ** can show TADF through the rISC between hot excitons, mainly within the *T*_2_–*S*_1_ and *T*_3_–*S*_2_ pairs. It is important to note that the *T*_2_–*S*_1_ rISC channel is thermodynamically allowed (*T*_2_ is higher in energy than *S*_1_ by 0.15 eV; thus, it does not require thermal activation). At the same time, the gaps *T*_2_–*T*_1_ and *T*_3_–*T*_1_ are relatively large (0.79 and 1.04 eV, respectively), preventing internal conversion within these pairs. For the related compound **PyPhPXZ**, the hot exciton rISC mechanism is similar to that of **PyPhDMAC**, but an additional *T*_2_ state is very close energetically to *S*_1_ (0.07 eV lower) and *T*_3_ (0.05 eV lower) states. This makes possible equilibration and mixing between *T*_2_ and *T*_3_ states through vibronic coupling, thus facilitating the rISC process. Similarly to **PyPhPTZ** and **PyPhDMAC**, the *T*_1_ state is very low-lying for **PyPhPXZ**. This allows us to predict slow *T*_2_–*T*_1_ and *T*_3_–*T*_1_ nonradiative deactivations. Summarizing, all of the studied compounds may be able to exhibit TADF through the rISC involving hot excitons, but deactivation of the *S*_1_ state through the ISC still dominates over the radiative decay of the *S*_1_ state. This results in low quantum yields of photoluminescence (PLQYs of the solid samples of **PyPhPXZ** and **PyPhPTZ** are less than 1%, PLQY of the layer of **PyPhCz** is just 2% and that of the layer **PyPhDMAC** is 4%). This, to a great extent, limits the applicability of the synthesized compounds for the preparation of active layers of OLEDs. Modification of the linking group and chemical tuning of donor moieties can be considered as potential ways to enhance radiative rate constants.

**Figure 3:**
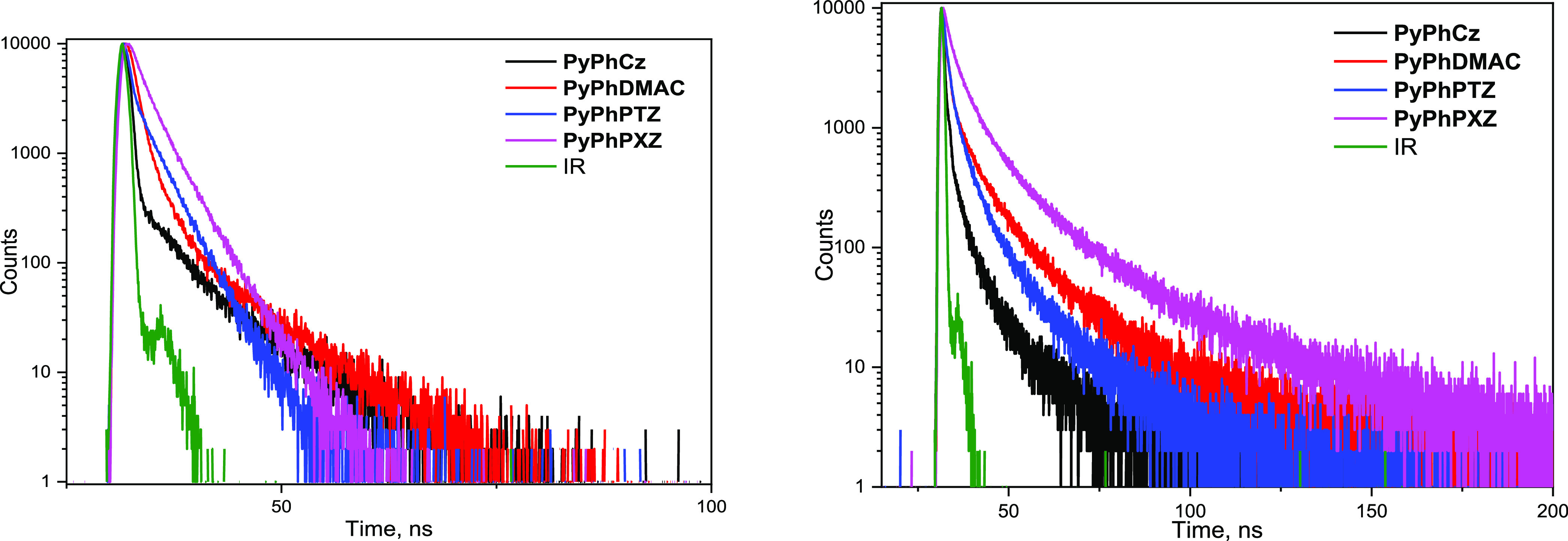
PL decay curves of the toluene solutions (left) and thin solid films (right) of the compounds recorded at room temperature.

Both the solutions and the solid samples of the compounds demonstrate similar trends of PL decay (Figure 3). The common trend for all of the cases is the presence of a very fast decay component in the range of 0.35–1.37 ns (Table 5). Accounting for the very weak fluorescence quantum yield observed for all of the compounds, this fast component can be assigned to nonradiative quenching of the *S*_1_ state most likely through the ISC channel. Indeed, for the S-containing compound **PyPhPTZ** sustaining internal heavy atom effect on the ISC process, fast decay component (τ_1_) is the fastest one in the whole series (just 0.34 ns for the solution in toluene). The second slower component of PL decay can be assigned to combined deactivation channels including ISC between *S*_1_ and particular low-lying *T_n_* states, prompt fluorescence, and TADF via a hot exciton channel. For example, one can assume that for **PyPhPXZ**, the toluene solution of which demonstrates the highest PLQY, τ_2_ corresponds to a large amount of prompt fluorescence combined with *S*_1_–*T*_2_ and *S*_1_–*T*_1_ ISC, but in the case of **PyPhPTZ**, τ_2_ lifetime corresponds mainly to *S*_1_–*T*_2_ and *S*_1_–*T*_1_ ISC accounting for negligible PLQY (Table 2).

**Table 5:** Fitting Results for PL Decay Curves of the Toluene Solutions and Thin Solid Films of the 2-Pyridone Derivatives at Room Temperature

	toluene			films		
compound	τ_1_, ns/Rel_1_, %^a^	τ_2_, ns/Rel_2_, %^a^	χ^2^	τ_1_, ns/Rel_1_, %^a^	τ_2_, ns/Rel_2_, %^a^	χ^2^
**PyPhCz**	0.42/94.76	6.75/5.24	1.279	0.55/97.15	7.57/2.85	1.293
**PyPhDMAC**	1.03/89.43	6.56/10.57	1.286	1.05/57.61	6.35/42.39	1.125
**PyPhPTZ**	0.35/42.50	2.65/57.50	1.151	0.57/95.71	6.54/4.29	1.278
**PyPhPXZ**	1.37/42.37	3.16/57.63	1.085	0.58/90.90	7.95/9.10	1.240

a Rel_1_ and Rel_2_ (in %) are contribution ratios of lifetimes τ_1_ and τ_2_, respectively

For the solid films, both τ_1_ and τ_2_ are generally lower than for toluene and the contribution of τ_1_ significantly dominates over τ_2_ (Table 5). Only for **PyPhDMAC**, the contribution of τ_2_ is equivalent to that of τ_1_. This observation we interpret in terms of hot exciton TADF combined with prompt fluorescence in line with conclusions by Kuehne^31^ and Ma^9,10^ that upper-level rISC in hot exciton TADF materials is fast and practically indistinguishable from fluorescence. In summary, the results of photoluminescence decay measurements confirm the presence of hot exciton TADF of **PyPhDMAC** and explain the unusually high efficiency of the corresponding sky-blue OLED.

### 2.4 Electrochemical and Photoelectrical Properties

**Figure 4:**
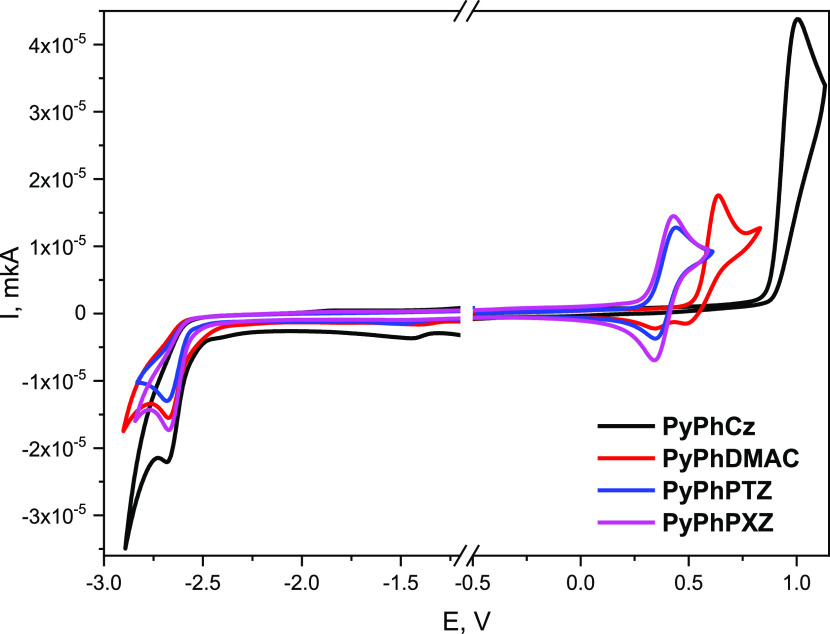
CV curves of the 2-pyridone-based derivatives.

Cyclic voltammetry (CV) measurements were used to estimate the ionization potentials (IP_CV_) and electron affinities (EA_CV_), which are the key parameters of the materials used for the fabrication of the OLEDs (Table 6). CV curves of the solutions of the compounds in anhydrous dimethylformamide (DMF) are shown in Figure 4. The positive and negative voltages were applied for the investigation of the redox behavior of the synthesized compounds and the estimation of the electronic energy levels. Due to the observation of both reduction and oxidation processes from CV curves of tested compounds, CV measurements of the solutions of the compounds revealed bipolar behavior. **PyPhDMAC**, **PyPhPTZ**, and **PyPhPXZ** containing 9,9-dimethyl-10*H*-acridine, 9*H*-phenothiazine, and 9*H*-phenoxazine moieties, respectively, were characterized by the quasi-reversible oxidation process, while compound **PyPhCz** with 9*H*-carbazole fragment showed the irreversible oxidation waves during the first scan, probably due to the participation of unsubstituted C-3 and C-6 positions of 9*H*-carbazole.^32^ The oxidation peaks of 2-pyridone derivatives were observed in the range of 0.28–0.88 V. Irreversible reduction processes with analogous shapes were observed for all of the studied compounds, due to the similar electron-deficient fragment. The reduction peaks were observed in the region of −2.52 to – 2.56 V. To estimate the IP_CV_ and EA_CV_ values, the onsets of oxidation and reduction potentials were used, respectively. The EA values were found to be quite similar for all of the investigated compounds (2.24–2.28 eV). The highest IP_CV_ value of 5.68 eV was observed for the compound **PyPhCz** containing the 9*H*-carbazole fragment. The lower IP_CV_ values (5.34, 5.12, and 5.08 eV) were obtained for other 2-pyridone-based derivatives. This observation can apparently be attributed to the stronger electron-donating effect of 9*H*-phenothiazine, 9*H*-phenoxazine, and 9,9-dimethyl-10*H*-acridane units.

**Table 6:** Electrochemical and Photoelectrical Characteristics of 2-Pyridone Derivatives^a^

compound	*E*_ox vs Fc^+^/Fc^onset^_	*E*_red vs Fc^+^/Fc^onset^_	IP_cv._, eV	EA_CV_, eV
**PyPhCz**	0.88	–2.53	5.68	2.27
**PyPhDMAC**	0.54	–2.52	5.34	2.28
**PyPhPTZ**	0.32	–2.55	5.12	2.25
**PyPhPXZ**	0.28	–2.56	5.08	2.24

a *E*_ox vs Fc^+^/Fc^onset^_, *E*_red vs Fc^+^/Fc^onset^_—onsets of oxidation and redaction potentials respectively; IP—ionization potential, IP_CV_ = *E*_ox_^onset^ + 4.8; EA—electron affinity, EA_CV_ = 4.8 – |*E*_red_^onset^|.

### 2.5 Electroluminescent Properties

Taking into account the highest solid fluorescence quantum yield and TADF effect, we utilized compound **PyPhDMAC** for the fabrication of nondoped and exciplex emission-based OLEDs. The nondoped device **A** has the following structure:
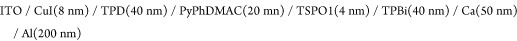


Devices **A**–**C** were fabricated by step-by-step deposition of hole- and electron transport layers, organic emissive layers, and metal electrodes onto precleaned ITO-coated glass substrate under a vacuum of 10^–5^ Torr. CuI^33^ and TPD^34^ (*N*,*N*′-bis(3-methylphenyl)-*N*,*N*′-diphenylbenzidine) were used as materials for hole-transporting layers. The TPBi^35^ 2,2′,2″-(1,3,5-benzinetriyl)-tris(1-phenyl-1-H-benzimidazole) and TSPO1 (diphenyl[4-(triphenylsilyl)phenyl]phosphine oxide)^36^ were used as electron-transporting and hole-blocking layers, respectively. The hole transport layer (HTL) and the electron transport layer (ETL) were chosen to confine holes and electrons in the emitting layer that improves the performance of the device. The Ca layer topped with a 200 nm aluminum (Al) layer was used as the cathode.^37,38^ The active area of the obtained devices was 2*3 mm^2^. The density–voltage and luminance–voltage dependences were recorded using a semiconductor parameter analyzer HP4145A. Electroluminescence (EL) spectra were recorded with an Ocean Optics USB2000 spectrometer.

As shown in Figure S4, the emission maxima wavelengths of device **A** were located in the range of 440–480 nm, which was close to the PL spectrum of the solid film of **PyPhDMAC**. This observation confirms exciton recombination in the emitting layer. The EL spectra were stable in the wide range of driving voltages, confirming the good color stability of the device (Figure S5a). Device **A** was characterized by relatively low-efficiency roll-off and good stability of efficiency of the device in all of the range of current density and by a maximum EQE value of 3.7% (Table 7).

**Table 7:** EL Characteristics of Devices **A**–**C**

device	*V*_on_, V	brightness^max^, Cd m^–2^	η_c_^max^, cd A^–1^	η_p_^max^, lm W^–1^	EQE^max^, %	λ_max_^EL^, nm	CIE_1976_ (u,v)
**A**	6	9900	6.1	2.3	3.7	482	(0.134, 0.391)
**B**	5.8	31 200	15.7	6.5	6.9	517	(0.121, 0.478)
**C**	5.7	35 370	16.1	6.9	9.8	425/480/519	(0.151, 0.441)

*V*_on_,—torn-on voltage, η_c_^max^—maximum current efficiency, η_p_^max^—maximum power efficiency, EQE_max_,—maximum value of external quantum efficiency, λ_max_^EL^—EL maximum, CIE1_976_ (*u*, *v*)—chromaticity coordinates.

**Figure 5:**
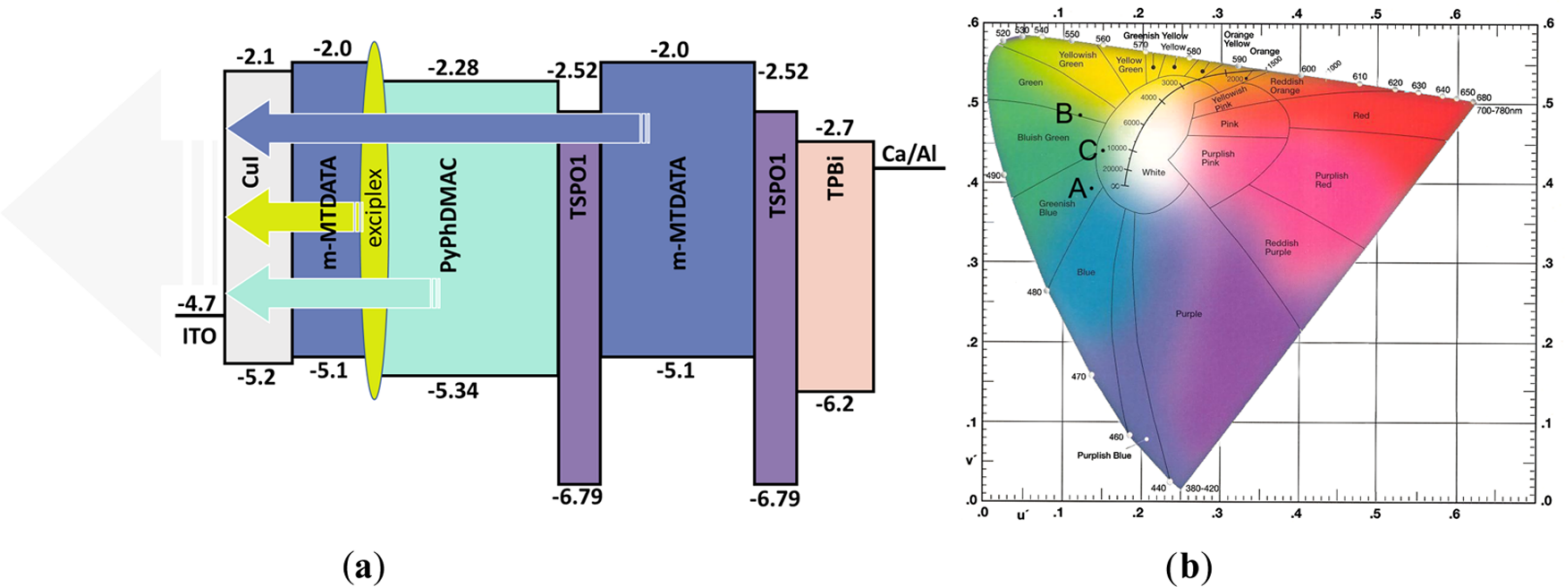
Schematic energy diagram of device **C** (a) and chromaticity diagram of devices **A**–**C** (b).

The exciplex emission-based OLED was prepared taking into account the results of the investigation of the photophysical properties of the **PyPhDMAC**. The 4,4′,4″-tris[phenyl(*m*-tolyl)amino]triphenylamine (*m*-MTDATA) was used as the donor counterpart for **PyPhDMAC** to induce exciplex emission. The energy difference of the HOMOs of *m*-MTDATA and **PyPhDMAC** is 0.24 eV, while that for the LUMO levels is 0.28 eV (Figure 5a). The structure of device **B** was as follows:
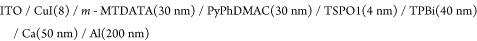


Figure S3b shows the relaxation dynamics of the exciplex emission of **PyPhDMAC** and *m*-MTDATA, which take longer times than the relaxation dynamics of fluorescence of solid films of **PyPhDMAC** or *m*-MTDATA. Because of the mismatch in the LUMO and HOMO energy levels, cross-coupling of the charge carriers occurred at the interface of the layers of *m*-MTDATA and **PyPhDMAC** and as a result, intense broad exciplex emission was observed in the region of 500–750 nm (Figure S3a). The spectrum of the exciplex of the mixture of *m*-MTDATA and **PyPhDMAC** is broader and red-shifted in comparison to the spectra of the solid films of **PyPhDMAC** or *m*-MTDATA (Figure S3a).

In order to fabricate the efficient WOLED, the technique of combining basic colors was used. The three-color WOLED was realized by simultaneously combining the intrinsic emission and exciplex emissions of the same materials (Figure 5a) similarly to previously reported results.^39,40^ The principal scheme of the fabricated WOLED based on exciplex enhanced TADF looks as follows:
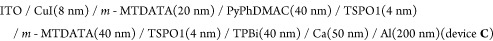


Since **PyPhDMAC** exhibited good performance in nondoped sky-blue OLEDs, it was anticipated that using **PyPhDMAC** as the blue-emitting compound, high-performance white OLED (WOLED) can be obtained. To accomplish this goal, the efficient yellow-green exciplex emission was introduced into the device to design full-color WOLED. Additionally, the thin layer of *m*-MTDATA was used to get blue emission. The layers of **PyPhDMAC** and *m*-MTDATA were separated by the layer of TSPO1 in the device to disturb the injection of carriers. The second TSPO1 interlayer with a thickness of 4 nm could block energy transfer between *m*-MTDATA and TPBi and modify the recombination zone. As a result, the designed structure of the device **C** allowed us to obtain exciton recombination zones from three emitters, i.e., deep-blue *m*-MTDATA, sky-blue **PyPhDMAC**, and yellowish-green interface exciplex emitters. The resulting WOLED presented a combined emission from different excited states, i.e., blue emission from the layer of *m*-MTDATA thin layer, exciplex emission from the interface of the layer of *m*-MTDATA and **PyPhDMAC** and sky-blue exciton emission from **PyPhDMAC** (Figure 5b).

**Figure 6:**
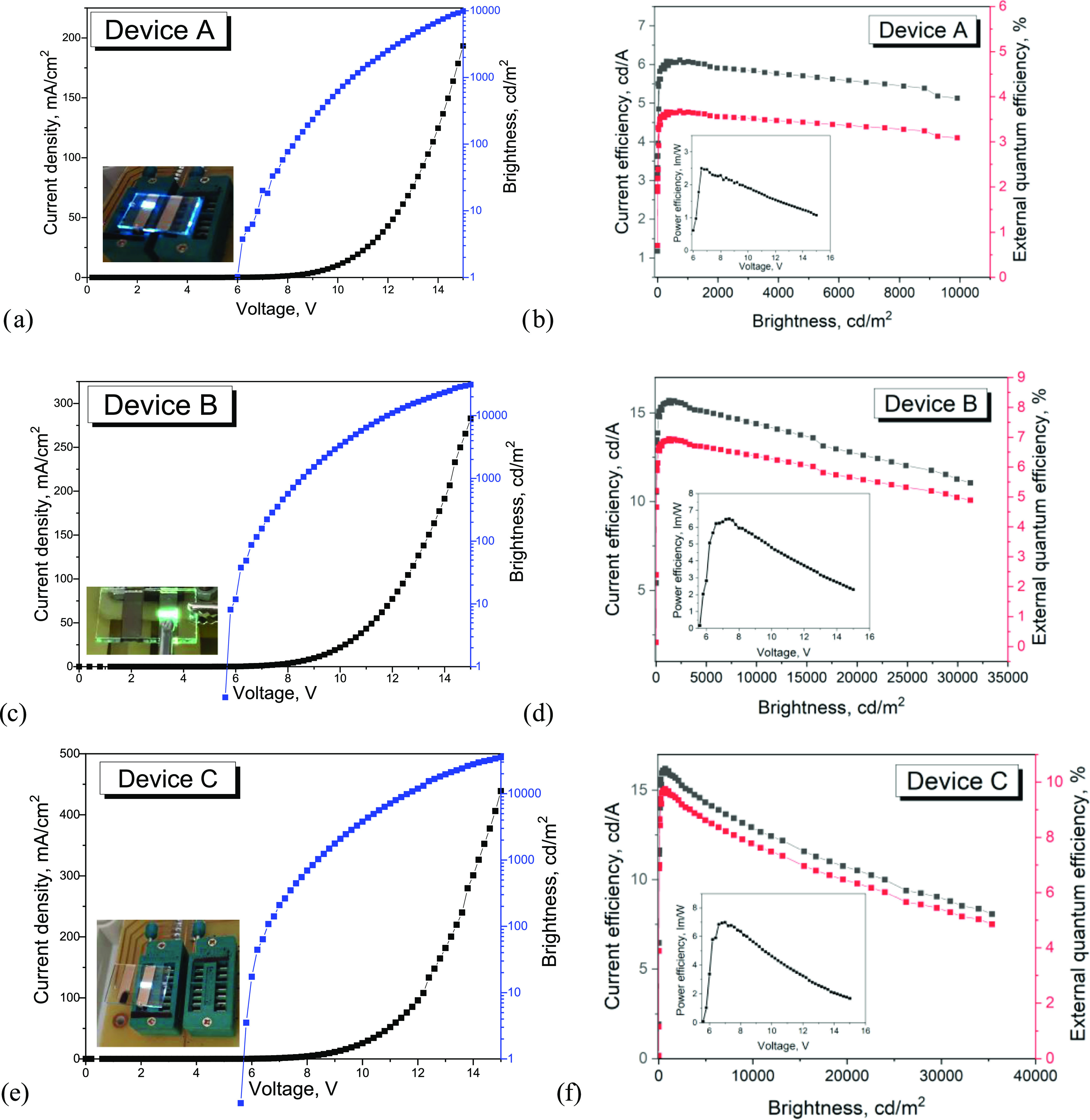
Current density and brightness versus voltage characteristics and photo of devices **A**–**C** (a, c, and e, respectively) and current efficiency-brightness-external quantum efficiency (b, d, and f, respectively) and power efficiency–voltage (inside) curves.

The lighting parameters are presented in Figures 6 and S6. Device **B**, based on exciplex emission of the interface of *m*-MTDATA and **PyPhDMAC** turned on at 5.8 V and showed high CE, PE, and EQE of 15.7 cd A ^–1^, 6.5 lm W ^–1^, and 6.9%, respectively (Table 7).

The graphs of the dependence of current efficiency and external quantum efficiency on brightness are shown in Figure 6. The external quantum efficiency was stable over the full scale of brightness and varied by 0.4% at luminance from 1000 to 10 000 Cd m^–2^. For device **B**, the value of quantum efficiency dropped by 1.7%, and for device **C**, it was two times smaller at a brightness of 35 000 Cd m^–2^ compared to those at 100 Cd m^–2^. On the one hand, these values are high for device **C**, but if we talk about operating voltages (1–10 V) and operating brightness (100–1000 Cd m^–2^), the decrease in performance is also within 0.5–2%. On the other hand, in the present work, the performance parameters of devices at critical values of voltages and current densities were studied. In device C (Figures 6 and S8), the current density is very high at maximum brightness, which causes thermal degradation effects in the device. This is due to several reasons: the thickness of the device, energy barriers between the emissive and functional layers, and the presence of nonradiative transitions.

The WOLED (device **C**) based on the combination of three deep-blue, sky-blue, and green-yellow emitters with CIE coordinates (*u*, *v*) of 0.151; 0.441 showed a turn-on voltage of 5.7 V, EQE exceeding 9.8%, and CE of more than 16 Cd A ^–1^.

## 3 Conclusions

The new series of donor–acceptor compounds based on 2-pyridone were synthesized, and their properties were investigated experimentally and theoretically. It was determined that compounds containing 9*H*-carbazole or 9,9-dimethyl-9,10-dihydroacridine moiety are crystalline materials while derivatives of 2-pyridone with 9*H*-phenothiazine or 9*H*-phenoxazine fragment are capable to form amorphous layers. The compounds exhibit emission in the violet and blue regions. The derivative of 2-pyridone and 9-dimethyl-9,10-dihydro-acridine is characterized by “hot” exciton TADF. Based on the theoretical calculations, it is assumed that a very large gap between *T*_2_ and *T*_1_ results in a slow internal conversion from *T*_2_ to *T*_1_. Therefore, the RISC process from *T*_2_ to *S*_1_ with a gap of only 0.01 eV is possible. Emissive TADF channel from *T*_2_ to *S*_1_ dominates over nonradiative quenching of “hot” excitons resulting in the external quantum efficiency of sky-blue OLED of 3.7%. The derivative containing a 9,9-dimethyl-9,10-dihydroacridine moiety also demonstrates exciplex-forming properties. The film of the compound and 4,4′,4″-tris[phenyl(*m*-tolyl)amino]triphenylamine shows an exciplex emission at 520 nm. The studied 2-pyridone derivative was tested in exciplex-based OLEDs exhibiting green-yellow and white emissions. The best white emitting device showed maximum current efficiency of 16.1 cd A^–1^, power efficiency of 16.9 lm W^–1^, and external quantum efficiency of 9.8%.

## 4 Experimental Section

### 4.1 Instrumentation

^1^H and ^13^C nuclear magnetic resonance (NMR) spectra were reordered on a Bruker DRX 400 P (400 MHz (^1^H), 100 MHz (^13^C)) spectrometer at room temperature in a deuterated chloroform (CDCl_3_) solution. The data are given as chemical shifts in δ (ppm), and tetramethylsilane (TMS) was used as an internal standard. Mass spectra were obtained by the electrospray ionization mass spectrometry (ESI-MS) method on an Esquire-LC 00084 mass spectrometer. Elemental analysis was performed with EuroEA Elemental Analyzer.

UV/vis spectra were recorded in quartz cells on an AvaSpec-USB2 spectrophotometer for 10^–5^ M solutions of the compounds. Photoluminescence (PL) spectra of 10^–5^ M solutions of the compounds were performed on an Edinburgh Instruments’ FLS980 fluorescence spectrometer. Thin solid films for recording UV/vis and PL spectra were prepared by spin-coating technique utilizing SPS-Europe Spin150 Spin processor using 2.5 mg/mL solutions of the compounds in THF on the precleaned quartz substrates. Photoluminescence quantum yields of the solutions and of the solid films were performed using the integrated sphere (Edinburgh Instruments) coupled to the FLS980 spectrometer, calibrated with two standards: quinine sulfate in 0.1 H_2_SO_4_ and rhodamine 6G in ethanol.^41^ Fluorescence decay curves of the samples were measured using a time-correlated single photon counting technique utilizing the PicoQuant PDL 820 ps diode laser as an excitation source (wavelength of 374 nm).

Differential scanning calorimetry (DSC) measurements were made on a TA Instruments “DSC Q100” calorimeter. The samples were heated at a scan rate of 10 °C/min under a nitrogen atmosphere. Thermogravimetric analysis (TGA) was performed on a “Mettler TGA/SDTA851e/LF/1100” at a heating rate of 20 °C/min under a nitrogen atmosphere. Electrochemical measurements were done using μAutolab Type III (EcoChemie, Netherlands) potentiostat, in a three-electrode cell using platinum rod as the counter electrode, glassy carbon as the working electrode (diameter 2 mm), and Ag/AgNO_3_ as the reference electrode with a scan rate of 2.5 mV/s with concentration of compounds 1.0 × 10^–4^ mol/dm^3^. The measurements were calibrated using internal standard ferrocene/ferrocenium (Fc/Fc^+^). Cyclic voltammetry (CV) experiments were conducted in the dry solvent solution containing 0.1 M tetrabutylammonium hexafluorophosphate (TBAPF_6_) as the electrolyte at room temperature under a nitrogen atmosphere. Deaeration of the solution was achieved by a nitrogen bubbling for ca. 10 min before measurement.

One of the synthesized derivatives was studied in electroluminescence devices. Devices were fabricated by vacuum deposition of organic semiconductor layers and metal electrodes onto the precleaned indium tin oxide (ITO)-coated glass substrate under vacuum of 10^–6^ Torr. ITO was used as the anode, which has great electric conductivity and light transmittance. Ca and Al are used as cathode. The HOMO energy level of CuI matches the structure with the weak potential barrier at the interface, which is conducive to hole injection and reducing the turn-on voltage, respectively. A hole-blocking layer (HBL) in the devices using TSPO1 is added between the emitting layer (*m*-MTDATA) and the electron-transporting layer, which blocks *m*-MTDATA and TPBi from forming exciplex at the interface. The density–voltage and luminance–voltage characteristics were measured by using a Keithley 6517 Binair instrument without passivation immediately after the preparation of the device. The brightness measurements were carried out by using a calibrated photodiode. The electroluminescence spectra were recorded with an Avaspec-2048L spectrometer. Device efficiencies were calculated from the luminance, current density, and EL spectrum.

### 4.2 Materials and Synthesis

The starting compounds, i.e., 2-hydroxypyridine, 1-bromo-4-iodobenzene, 9,10-dihydroacridine, 1,10-phenanthroline, palladium acetate (Pd(CH_3_COO)_2_), tri*tert*-butylphosphine (P(*t*-Bu)_3_), copper, copper(I) chloride (CuCl), cesium carbonate (Cs_2_CO_3_), potassium carbonate (K_2_CO_3_), and sodium chloride (NaCl), were purchased from Sigma-Aldrich and used as received.

#### 4.2.1 1-(4-Bromophenyl)pyridin-2(1*H*)-one (**BrPh2PY**)

BrPh2PY was synthesized by Ullmann condensation.^42^ 1-Bromo-4-iodobenzene (7.45 g, 0.026 mol), 2-hydroxypyridine (2.5, 0.026 mol), copper (0.168 g, 2.6 mmol), copper(I) chloride (0.26 g, 2.6 mmol), potassium carbonate (7.26 g, 0.52 mol), and 1,10-phenanthroline (0.974 g, 3.4 mmol) were dissolved in dimethyl sulfoxide (DMSO) under argon atmosphere. The reaction mixture was heated at 140 °C for 24 h. After cooling, the reaction mixture was filtered through a 2 cm layer of Celite and washed with ethyl acetate. The solvent was removed under reduced pressure, and the product was recrystallized from isopropanol and vacuum-dried to afford light-brown crystals. ^1^H NMR (400 MHz, CDCl_3_) δ 7.55 (d, *J* = 8.3 Hz, 2H), 7.38–7.30 (m, 1H), 7.22 (t, *J* = 8.9 Hz, 3H), 6.62 (d, *J* = 9.3 Hz, 1H), 6.20 (t, *J* = 6.7 Hz, 1H). ^13^C NMR (100 MHz, CDCl_3_) δ: 162.21, 140.18, 139.78, 138.55, 137.50, 132.57, 128.33, 121.95, 106.44. Mp 100 °C.

#### 4.2.2 1-(4-(9*H*-Carbazol-9-yl)phenyl)pyridin-2(1*H*)-one (**PyPhCz**)

**PyPhCz** was prepared by Ullmann condensation.^42^ A mixture of BrPh2PY (0.5 g, 2.00 mmol), 9*H*-carbazole (0.4 g, 2.39 mmol), Cu (0.01 g, 0.20 mmol), CuCl (0.02 g, 0.20 mmol), K_2_CO_3_ (0.55 g, 3.98 mmol), and 1,10-phenanthroline (0.07 g, 0.40 mmol) were dissolved in *o*-DCB (10 mL) under an argon atmosphere. The reaction mixture was heated at 170 °C for 24 h. After cooling down, the reaction mixture was filtrated through a 2 cm layer of Celite and washed with dichloromethane (DCM). The solvent was removed under reduced pressure, and the crude product was purified by flash column chromatography on silica gel using an eluent system EtOAc/HEX = 1.5/1, crystallized from isopropanol and vacuum-dried to afford yellow crystals (0.58 g, 87% yield). ^1^H NMR (400 MHz, CDCl_3_) δ 8.08 (d, *J* = 7.8 Hz, 2H), 7.64 (d, *J* = 8.6 Hz, 2H), 7.57 (d, *J* = 8.6 Hz, 2H), 7.44–7.33 (m, 6H), 7.24 (t, *J* = 7.4 Hz, 2H), 6.65 (d, *J* = 8.9 Hz, 1H), 6.25 (t, *J* = 6.7 Hz, 1H). ^13^C NMR (100 MHz, CDCl_3_) δ: 162.40, 140.58, 140.08, 139.55, 137.82, 128.14, 127.67, 126.13, 123.59, 122.15, 120.36, 109.78, 106.32. Elemental analysis calcd for C_23_H_16_N_2_O (%): C 82.12, H 4.79, N 8.33, O 4.76; found (%): C 82.06, H 4.77, N 8.39. *m*/*z*: 336.13.

#### 4.2.3 1-(4-(9,9-Dimethylacridin-10(9*H*)-yl)phenyl)pyridin-2(1*H*)-one (**PyPhDMAC**)

**PyPhDMAC** was prepared by the Buchwald–Hartwig cross-coupling reaction.^43^ A mixture of appropriate BrPh2PY (0.5 g, 2.00 mmol), 9,9-dimethyl-10*H*-acridane (0.5 g, 2.39 mmol), and Cs_2_CO_3_ (1.30 g, 4.00 mmol) was dissolved under argon in dry toluene (10 mL) and was stirred for 10 min at room temperature. Then, Pd(CH_3_COO)_2_ (0.09 g, 0.40 mmol) and P(*t*-Bu)_3_ (0.04 g, 0.19 mmol) under argon were added. The reaction mixture was heated at 100 °C for 24 h. After cooling down, the reaction mixture was filtrated through a 2 cm layer of Celite and washed with DCM. After that, the solvent was removed under reduced pressure. The crude product was purified by flash column chromatography on silica gel using eluent system EtOAc/HEX = 1.5/1, crystallized from isopropanol, and vacuum-dried to afford olive crystals (0.33 g, 45% yield). ^1^H NMR (400 MHz, CDCl_3_) δ 7.61 (d, *J* = 8.5 Hz, 2H), 7.45–7.34 (m, 6H), 6.96–6.84 (m, 4H), 6.65 (d, *J* = 9.2 Hz, 1H), 6.25 (dd, *J* = 11.0, 7.4 Hz, 3H), 1.62 (s, 6H). ^13^C NMR (100 MHz, CDCl_3_) δ: 162.29, 141.29, 140.60, 140.05, 137.79, 132.29, 130.14, 129.08, 126.45, 125.31, 122.18, 120.84, 114.17, 106.27, 35.99, 31.25. Elemental analysis calcd for C_26_H_22_N_2_O (%): C 82.51, H 5.86, N 7.40, O 4.23; found (%): C 82.46, H 5.91, N 7.45. *m*/*z*: 378.17.

#### 4.2.4 1-(4-(10*H*-Phenothiazin-10-yl)phenyl)pyridin-2(1*H*)-one (**PyPhPTZ**)

**PyPhPTZ** was prepared by the same method as **PyPhDMAC**, starting from BrPh2PY (0.5 g, 2.00 mmol), phenothiazine (0.29 g, 2.44 mmol), Cs_2_CO_3_ (1.30 g, 4.00 mmol), Pd(CH3COO)_2_ (0.09 g, 0.40 mmol), and P(*t*-Bu)_3_ (0.04 g, 0.19 mmol) in dry toluene (10 mL). The crude product was purified by flash column chromatography on silica gel using an eluent system EtOAc/HEX = 1.5/1, crystallized from isopropanol, and vacuum-dried to afford white crystals (0.31 g, 43% yield). ^1^H NMR (400 MHz, CDCl_3_) δ 7.51 (d, *J* = 8.6 Hz, 2H), 7.39 (d, *J* = 8.7 Hz, 2H), 7.37–7.33 (m, 2H), 7.03 (dd, *J* = 7.4, 1.4 Hz, 2H), 6.85 (dt, *J* = 24.7, 7.2 Hz, 4H), 6.65–6.60 (m, 1H), 6.39 (d, *J* = 8.0 Hz, 2H), 6.22 (t, *J* = 6.7 Hz, 1H). ^13^C NMR (100 MHz, CDCl_3_) δ: 162.33, 140.02, 139.46, 137.78, 129.34, 128.66, 127.09, 123.29, 122.63, 122.11, 117.97, 106.25. Elemental analysis calcd for C_23_H_16_N_2_OS (%): C 74.98, H 4.38, N 7.60, O 4.34, S 8.70; found (%): C 74.92, H 4.33, N 7.66, S 8.75. *m*/*z*: 368.10.

#### 4.2.5 1-(4-(10*H*-Phenoxazin-10-yl)phenyl)pyridin-2(1*H*)-one (**PyPhPXZ**)

**PyPhPXZ** was prepared by the same method as **PyPhDMAC**, starting from BrPh2PY (0.5 g, 2.00 mmol), phenoxazine (0.44 g, 2.40 mmol), Cs_2_CO_3_ (1.30 g, 4.00 mmol), Pd(CH_3_COO)_2_ (0.09 g, 0.40 mmol), and P(*t*-Bu)_3_ (0.04 g, 0.19 mmol) in dry toluene (10 mL). The crude product was purified by flash column chromatography on silica gel using eluent system EtOAc/HEX = 1.5/1, crystallized from isopropanol, and vacuum-dried to afford light-brown crystals (0.31 g, 45% yield). ^1^H NMR (400 MHz, CDCl_3_) δ 7.57 (d, *J* = 8.5 Hz, 2H), 7.44–7.33 (4H), 6.66–6.52 (m, 8H), 5.94 (dd, *J* = 7.8, 1.3 Hz, 2H). ^13^C NMR (100 MHz, CDCl_3_) 162.23, 143.94, 140.70, 140.10, 139.08, 137.62, 134.00, 131.86, 129.29, 123.31, 122.17, 121.66, 115.58, 113.44, 106.35. Elemental analysis calcd for C_23_H_16_N_2_O_2_ (%): C 78.39, H 4.58, N 7.95, O 9.08; found (%): C 78.44, H 4.04, N 7.89. *m*/*z*: 352.12.

### 4.3 Computational Details

Ground singlet state (*S*_0_) of **PyPhCz**, **PyPhDMAC**, **PyPhPTZ**, and **PyPhPXZ** molecules were optimized at the B3LYP/6-31G(d)^44−47^ level of density functional theory (DFT) using Grimme’s empirical dispersion correction (GD3).^48^ Based on optimized *S*_0_ state geometries, the first singlet excited state (*S*_1_) geometry was optimized by time-dependent (TD) DFT method^49^ employing the same GD3-B3LYP/6-31G(d) approach, while the first triplet excited state (*T*_1_) was optimized by spin-unrestricted UB3LYP/6-31G(d) method. The polarizable continuum model (PCM) was used during all geometry optimization procedures.^50^ By using optimized *S*_1_ and *T*_1_ geometries, the energies of singlet and triplet excited states were clarified by range-separated LC-ωPBE^51^ with manually tuned ω value equal to 0.14 that gives the best agreement with experimentally observed fluorescence wavelength. All of these calculations were performed within Gaussian16 software.^52^ The spin–orbit coupling matrix elements ⟨*S*_1_|**Ĥ**_**SO**_|*T*_*n*_⟩ were calculated by using zeroth-order regular approximation (ZORA)^53^ for **Ĥ**_**SO**_ operator at TDDFT/PBE0/TZP^54,55^ level of theory with accounting of solvent effect within COSMO model^56^ similarly to the methodology proposed by Brédas et al.^57^ SOC calculations were performed within ADF2021 software.^58^

## Supporting Information Available

Thermal properties (DSC curves), photophysical properties of the studied materials and electroluminescence properties of the fabricated devices, and ^1^H and ^13^C spectra of synthesized materials (PDF)
