# Supplementary material for: Derivatives of 2-Pyridone Exhibiting Hot-Exciton TADF for Sky-Blue and White OLEDs
Source: ACS Appl Electron Mater. 2023 Jul 21;5(8):4174–86. doi: 10.1021/acsaelm.3c00443 (PMC10449007; doi:10.1021/acsaelm.3c00443)

Supporting Information

Derivatives of 2-pyridone exhibiting hot-exciton TADF for sky-blue and white OLEDs

Iryna Danyliv[a], Khrystyna Ivaniuk[a], Yan Danyliv[a], Igor Helzhynskyy[a], Viktorija Andrulevicienė[b], Dmytro Volyniuk[b], Pavlo Stakhira[a], Glib V. Baryshnikov[c,d*], Juozas V. Grazulevicius[b*]

[a] *Lviv Polytechnic National University, Stepan Bandera 12, 79013, Lviv, Ukraine*

[b] *Department of Polymer Chemistry and Technology, Kaunas University of Technology, K. Barsauskas str. 59, Kaunas 51423, Lithuania*

[c] *Laboratory of Organic Electronics, Department of Science and Technology, Linköping University, SE-60174 Norrköping, Sweden*

[d] *Department of Chemistry and Nanomaterials Science, Bohdan Khmelnytsky National University, 18031 Cherkasy, Ukraine*

[*] Corresponding authors. E-mail address: glib.baryshnikov@liu.se (Glib Baryshnikov)

E-mail address: juozas.grazulevicius@ktu.lt (Juozas Vidas Grazulevicius)

Thermal properties

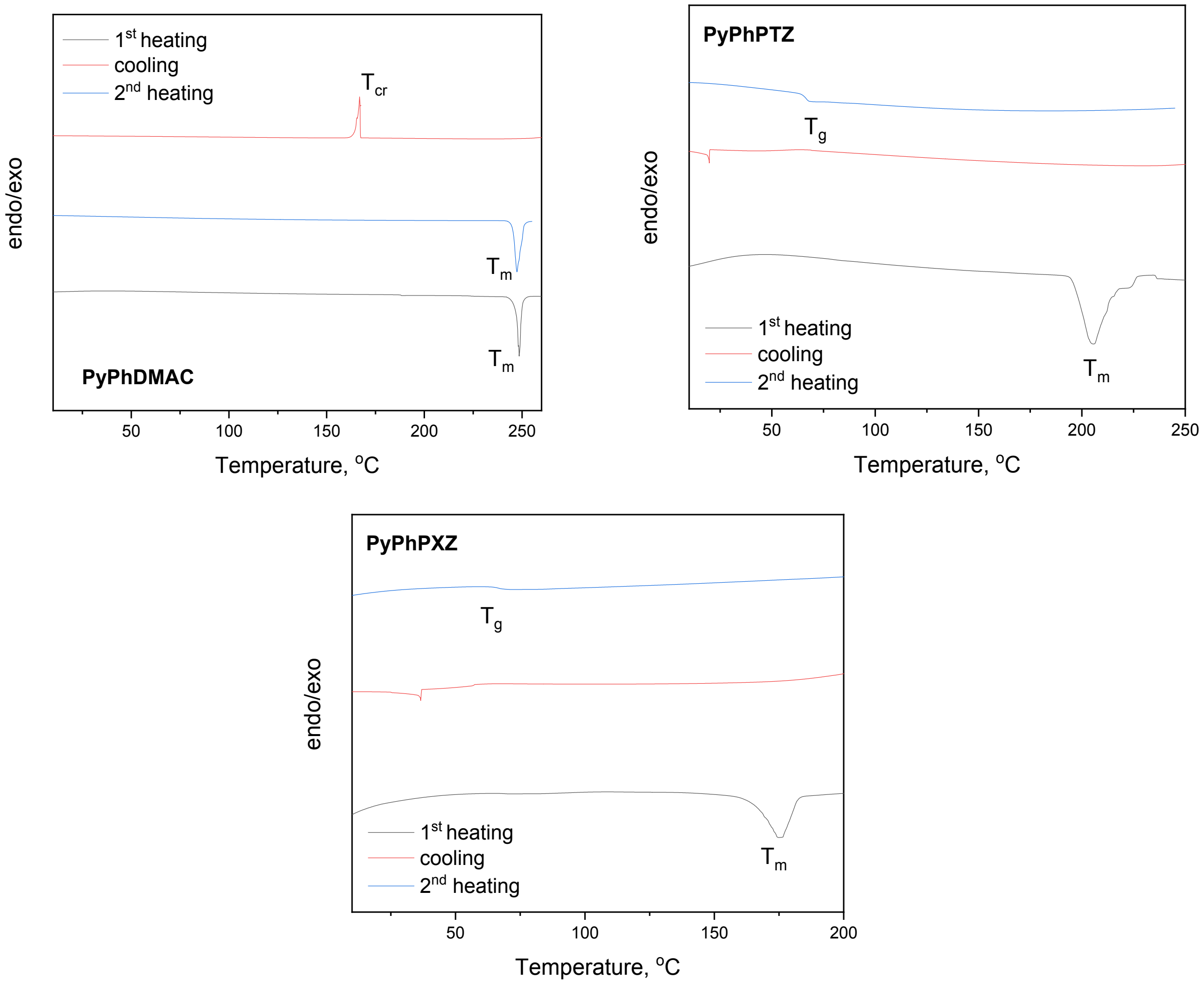

Figure S1. DSC curves of the derivatives of 2-pyridone.

Frontier molecular orbitals

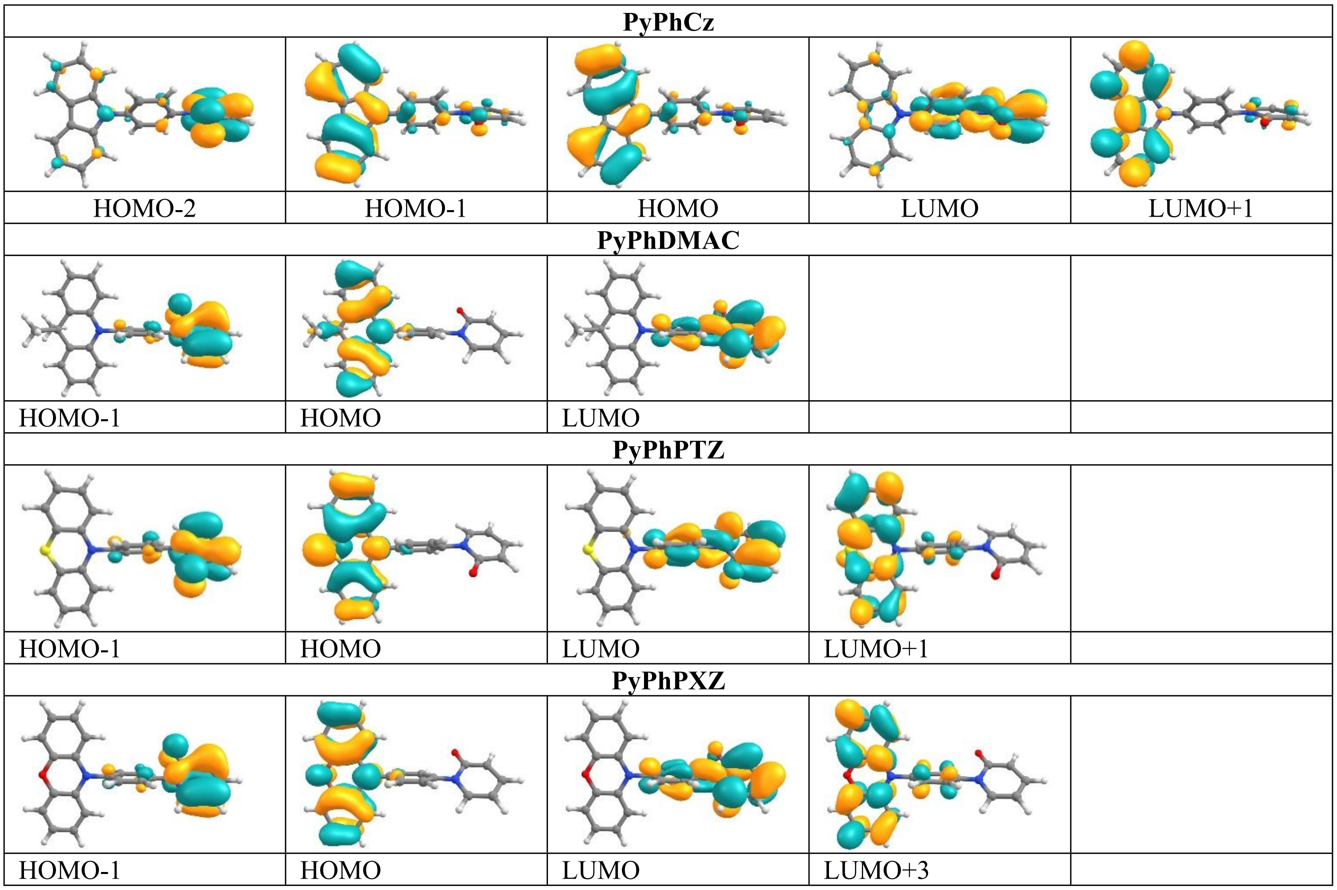

Figure S2. Selected molecular orbitals forming the low-lying excited states for the studied emitters (calculated at T1 state geometry and correspond to the last column in Table 4).

Photophysical properties

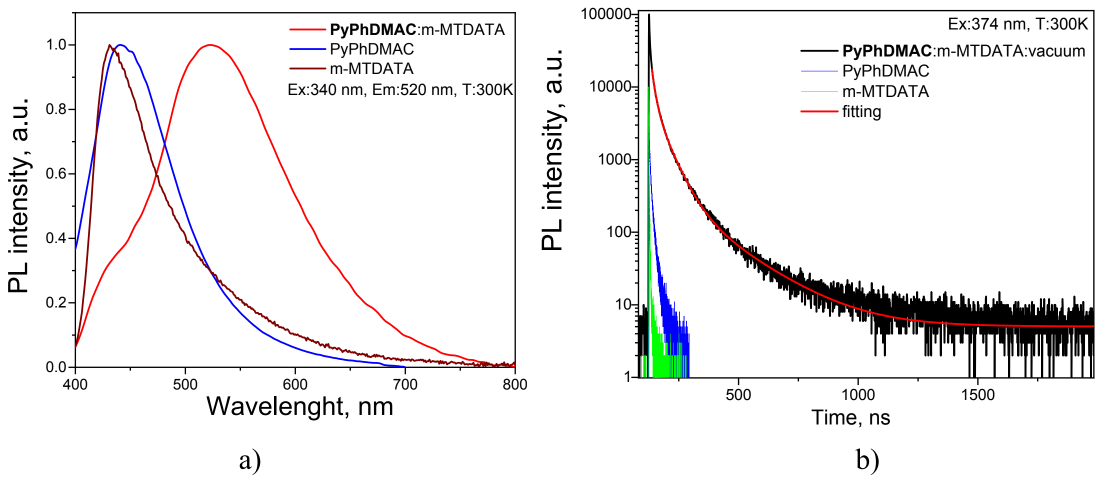

Figure S3. PL spectra (a) and PL decay curve (b) of the solid films of the molecar mixture of **PyPhDMAC** and **m-MTDATA, PyPhDMAC** and **m-MTDATA**.

Table S1. PL decay data of the of the films of **PyPhDMAC:m-MTDATA.**

Param.	Value/ns	Std. Dev./ns	Param	Value/ns	Std. Dev./ns	Rel.%
τ_1	13.22	0.16	B1	12394.88	93.30	30.89
τ_2	50.83	0.56	B2	5784.92	86.27	55.44
τ_3	186.08	2.98	B3	389.57	15.48	13.67

1. Electroluminescence properties

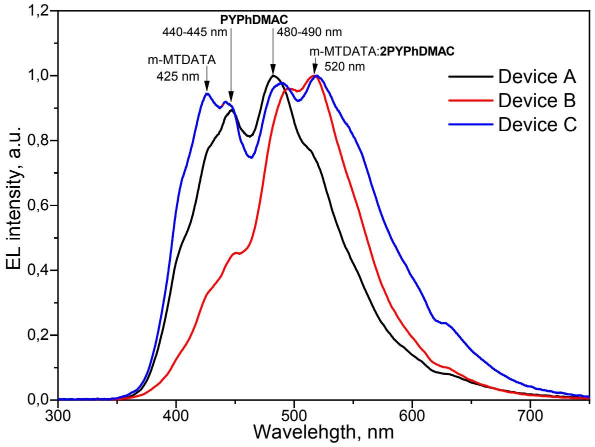

Figure S4. Electroluminescence spectra of **A**, **B** and **C** devices

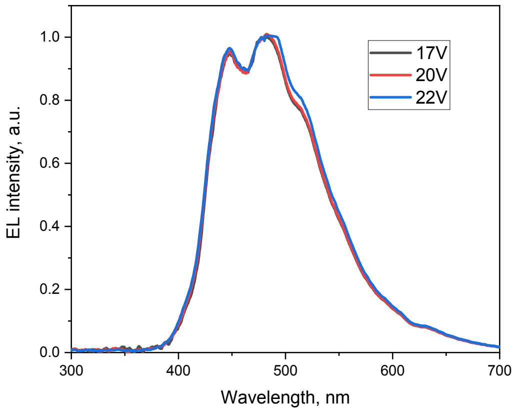

a)

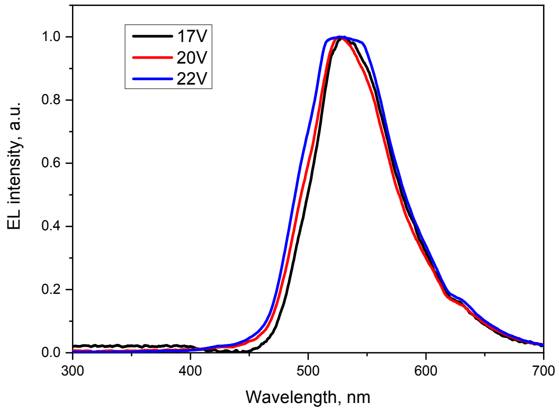

b)

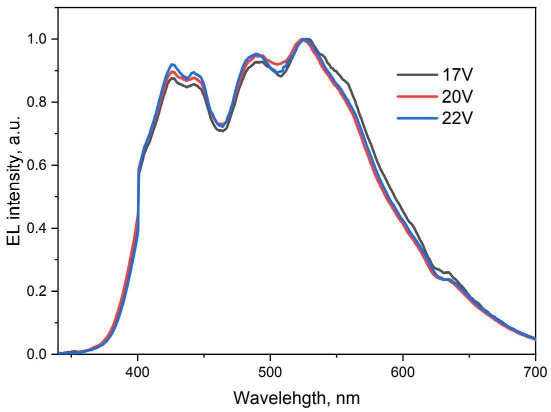

c)

Figure S5. Electroluminescence spectra of the Devices A (a), B (b) and C (c) at different voltages (17, 20 and 22 V).

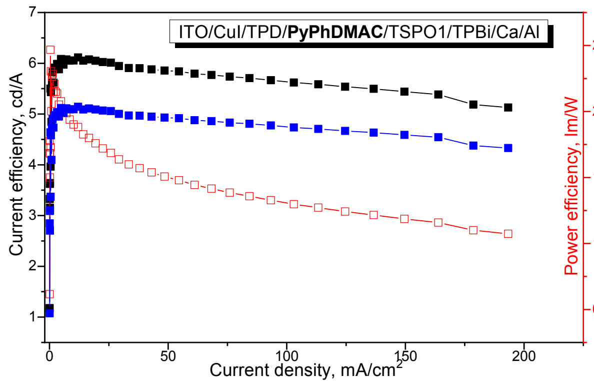

a)

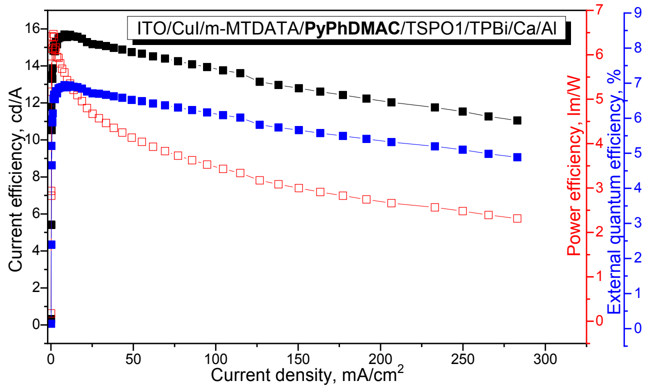

b)

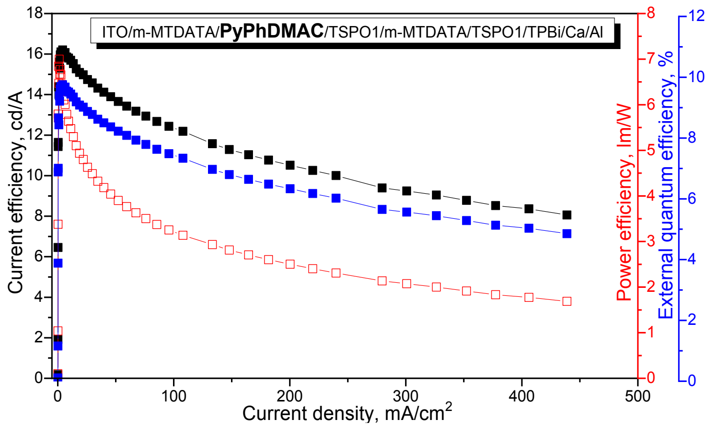

c)

Figure. S6. Current density-voltage and luminance-voltage, power efficiency – current density, current efficiency - current density and external quantum efficiency – current density of the devices **A** (a), **B**(b) and **C** (c).

^{1}H and ^{13}C spectra of PyPhCz

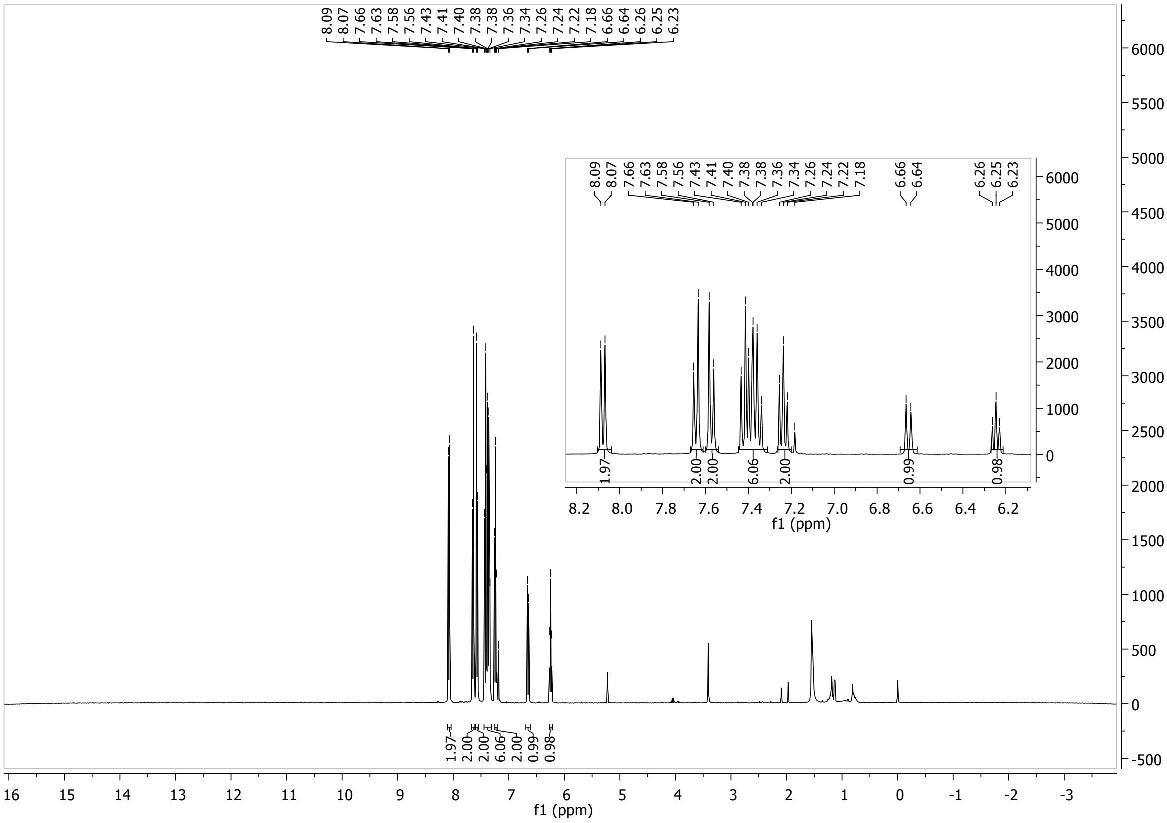

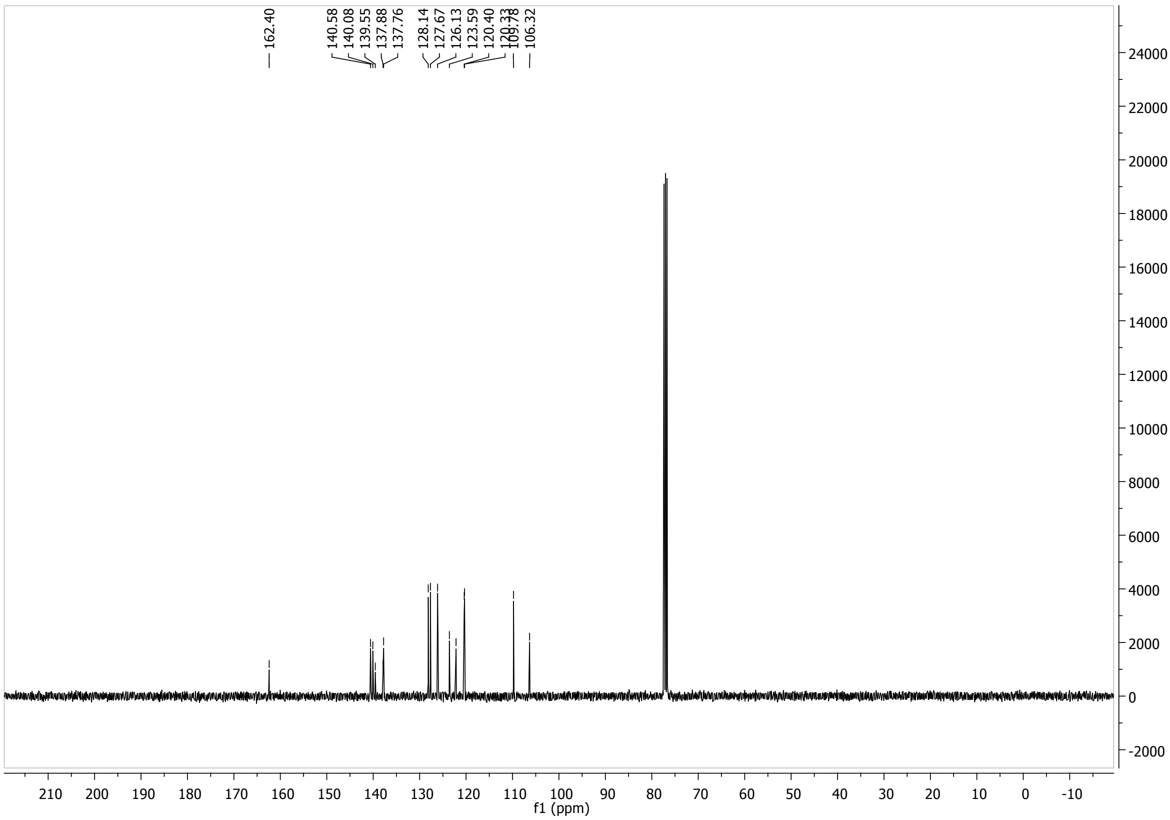

^{1}H and ^{13}C spectra of PyPhDMAC

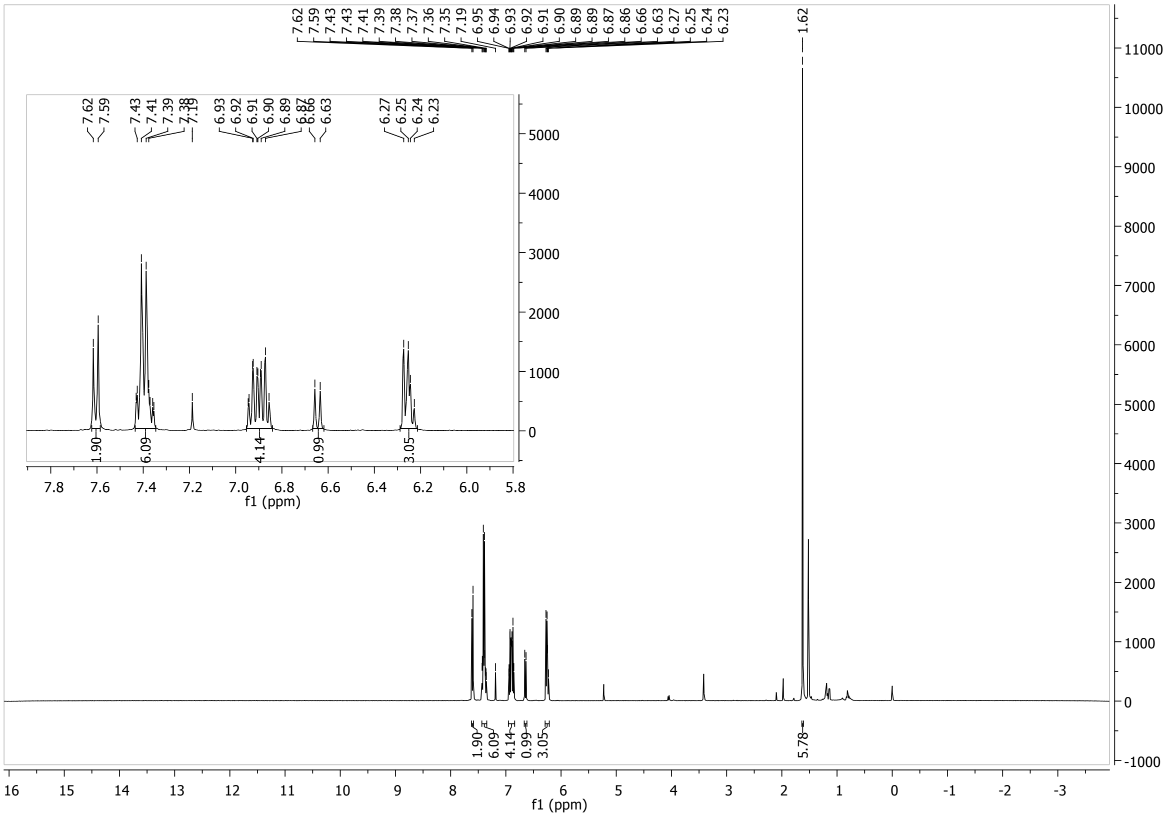

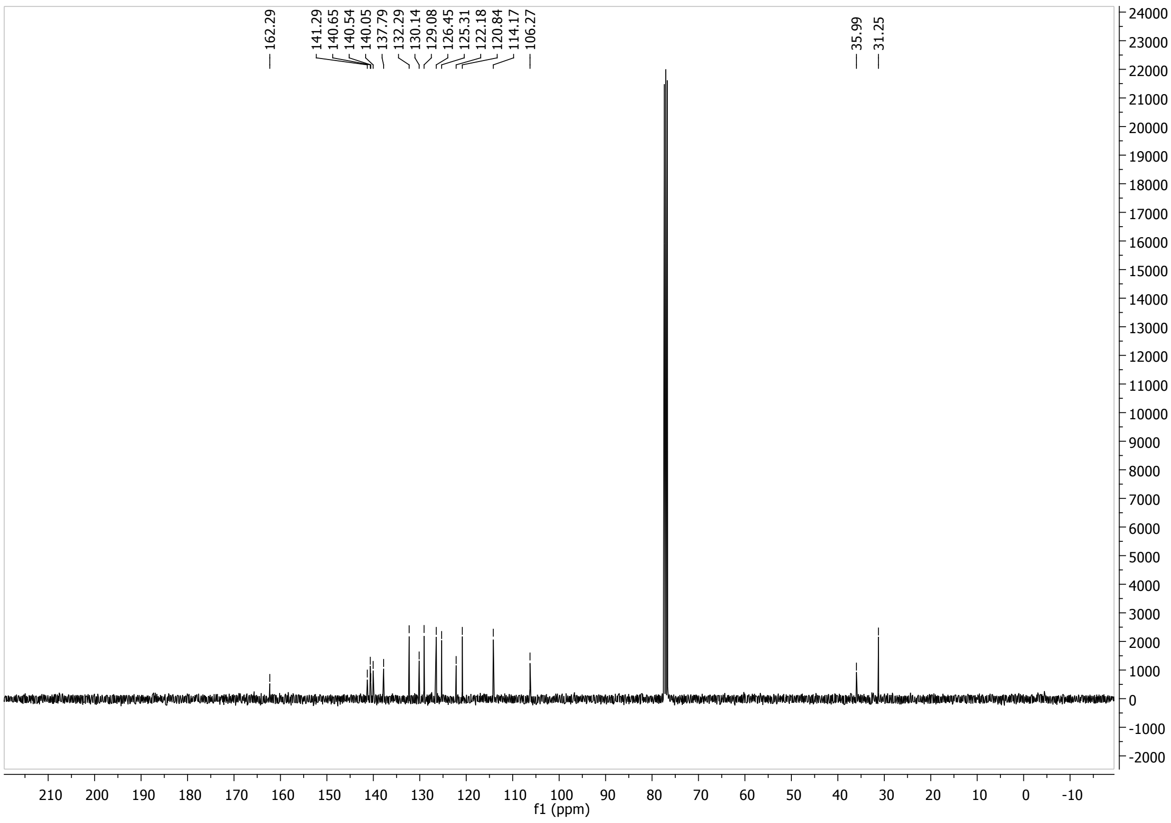

^{1}H and ^{13}C spectra of PyPhPTZ

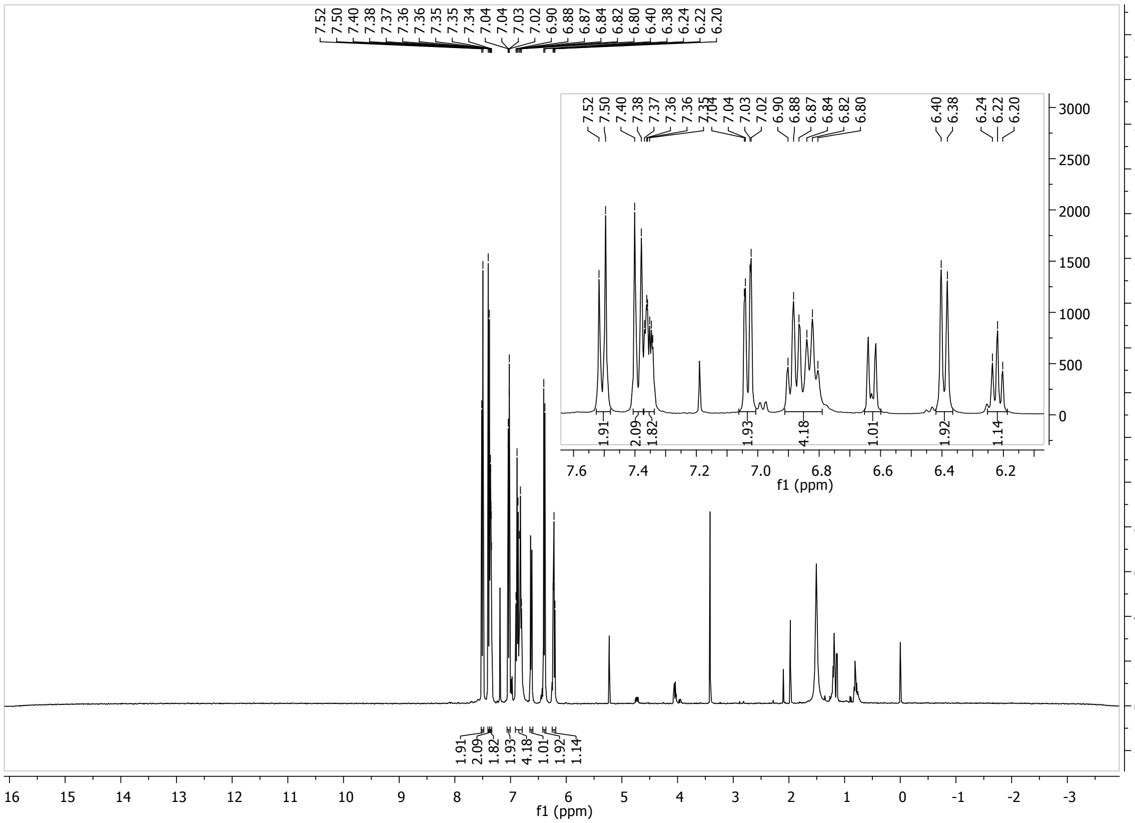

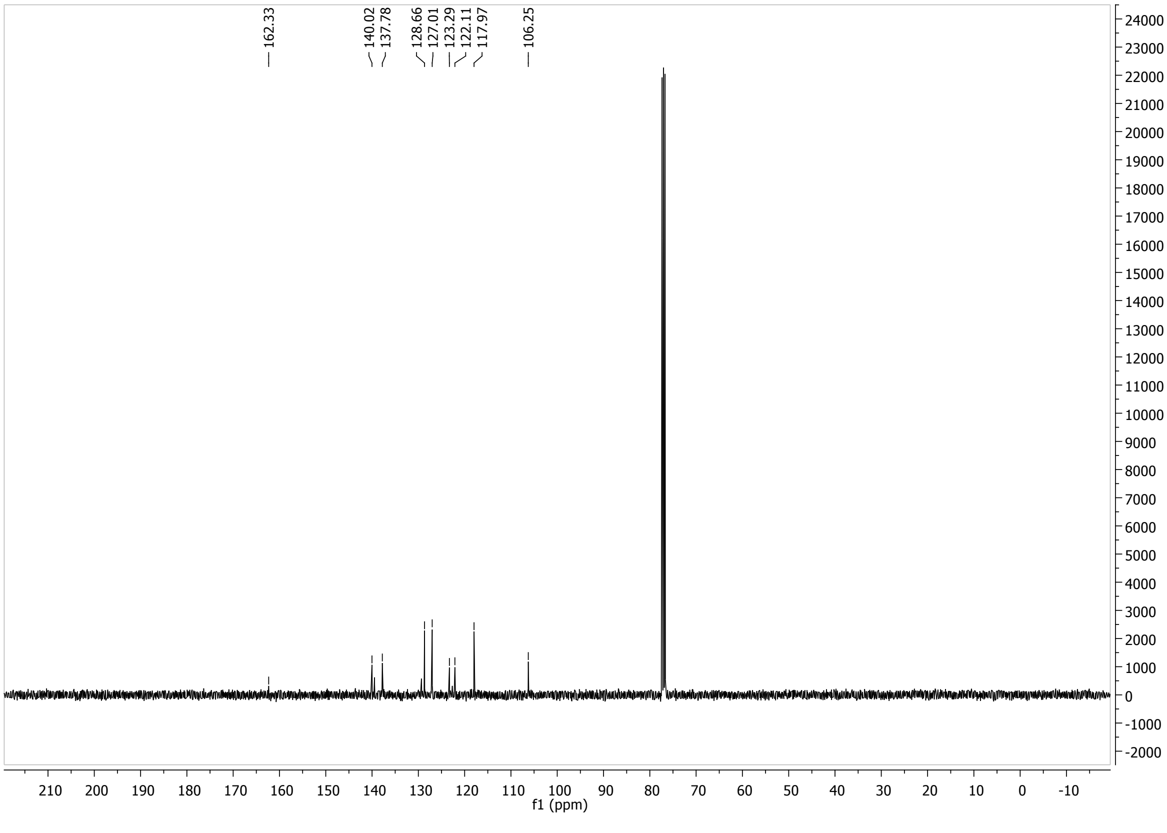

^{1}H and ^{13}C spectra of PyPhPXZ

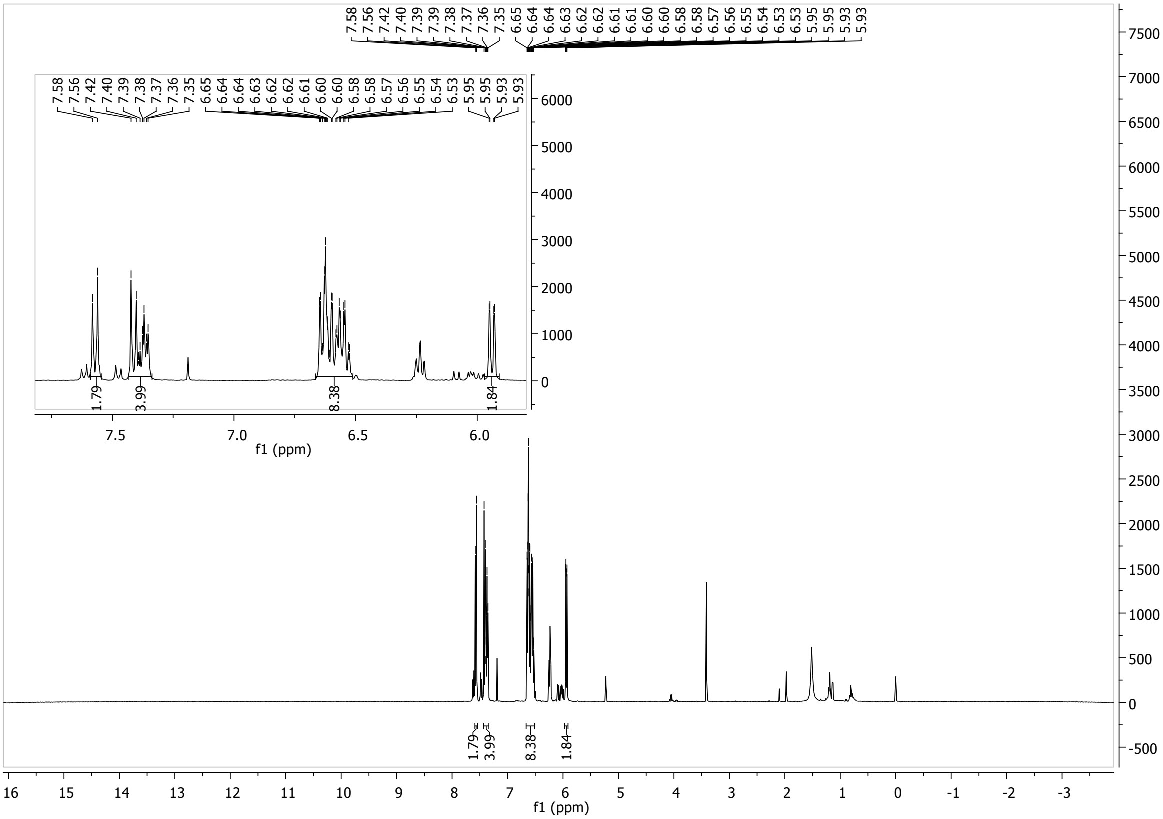

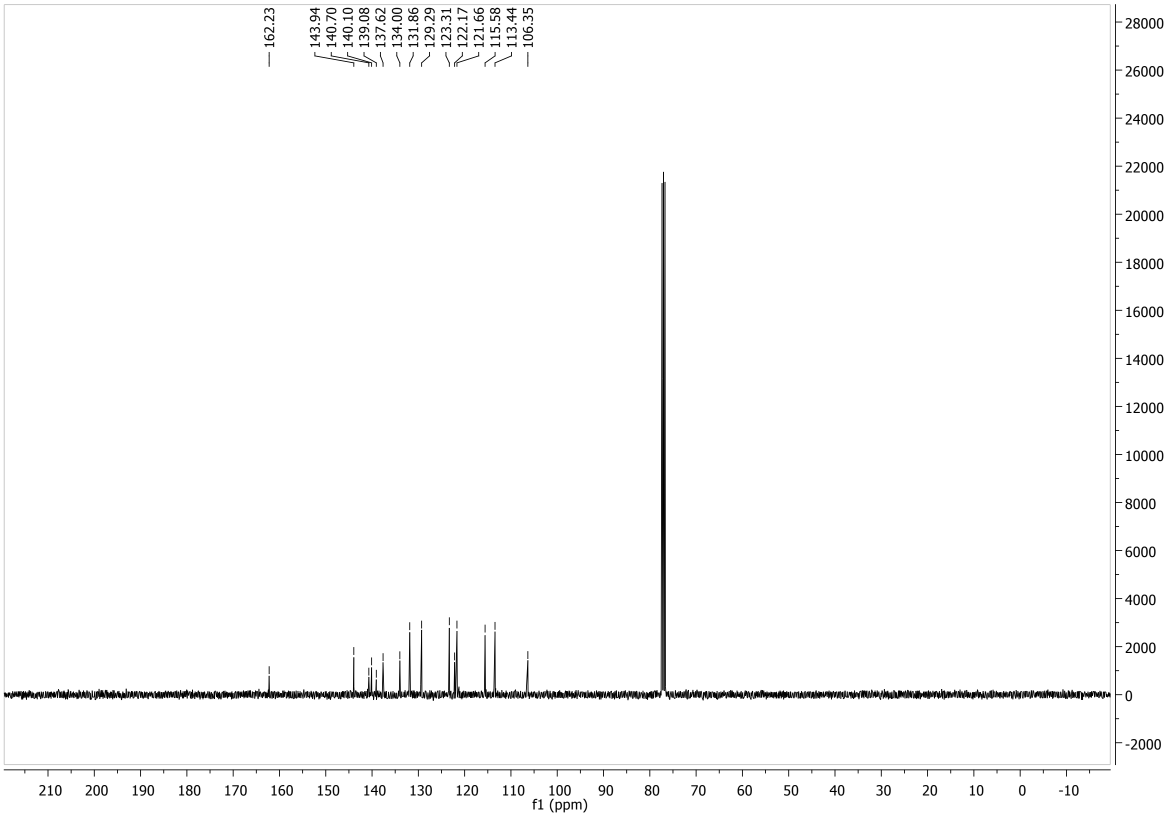

Supplement: Supplementary file 1 — el3c00443_si_001.pdf [file el3c00443_si_001.pdf]
